# Rewiring of the inferred protein interactome during blood development studied with the tool PPICompare

**DOI:** 10.1186/s12918-017-0400-x

**Published:** 2017-04-04

**Authors:** Thorsten Will, Volkhard Helms

**Affiliations:** 1 Center for Bioinformatics, Saarland University, Campus E2.1, Saarbrücken, 66123 Germany; 2 Graduate School of Computer Science, Saarland University, Campus E1.3, Saarbrücken, 66123 Germany

**Keywords:** Protein-protein interaction networks, Alternative splicing, Differential network analysis, Hematopoiesis

## Abstract

**Background:**

Differential analysis of cellular conditions is a key approach towards understanding the consequences and driving causes behind biological processes such as developmental transitions or diseases. The progress of whole-genome expression profiling enabled to conveniently capture the state of a cell’s transcriptome and to detect the characteristic features that distinguish cells in specific conditions. In contrast, mapping the physical protein interactome for many samples is experimentally infeasible at the moment. For the understanding of the whole system, however, it is equally important how the interactions of proteins are rewired between cellular states. To overcome this deficiency, we recently showed how condition-specific protein interaction networks that even consider alternative splicing can be inferred from transcript expression data. Here, we present the differential network analysis tool PPICompare that was specifically designed for isoform-sensitive protein interaction networks.

**Results:**

Besides detecting significant rewiring events between the interactomes of grouped samples, PPICompare infers which alterations to the transcriptome caused each rewiring event and what is the minimal set of alterations necessary to explain all between-group changes. When applied to the development of blood cells, we verified that a reasonable amount of rewiring events were reported by the tool and found that differential gene expression was the major determinant of cellular adjustments to the interactome. Alternative splicing events were consistently necessary in each developmental step to explain all significant alterations and were especially important for rewiring in the context of transcriptional control.

**Conclusions:**

Applying PPICompare enabled us to investigate the dynamics of the human protein interactome during developmental transitions. A platform-independent implementation of the tool PPICompare is available at https://sourceforge.net/projects/ppicompare/.

**Electronic supplementary material:**

The online version of this article (doi:10.1186/s12918-017-0400-x) contains supplementary material, which is available to authorized users.

## Background

Generally, every apparatus is better specified by the connection of its parts than by the sole list of parts. In the same way, the state of a cell is better described by the cooperative action of its active molecular machinery than by a simple list of its genome-encoded building blocks. Consequently, decades of research have gone into detecting physical interactions between proteins. Aggregating all this effort into comprehensive protein-protein interaction networks (PPINs) that represent the known protein interactome of an organism has been an important achievement [1, 2].

However, a static representation of the full interactome does not reflect its wiring in different tissues, cell types, diseases or any other arbitrary cellular state. Experimental data on protein-protein interactions (PPIs) in particular contexts is very limited and it is unclear whether its amount will increase substantially in the near future [1, 3]. Previous experimental studies typically focused on very specific issues, such as the perturbation of individual interactions by disease mutations [4, 5] or posttranslational modifications [6], and covered only small subsets of the proteome. The general lack of context-sensitive interactome data is commonly overcome by computational methods that integrate condition-specific gene expression data with the known PPIN of that organism so that at least the influence of that factor is considered on a genome-wide scale. A straightforward approach is to filter the PPIs to the protein-coding genes that are expressed in a certain condition. This strategy was applied before in the contexts of tissues and cell types [7–10] and of diseases [11–13].

The aforementioned limitation concerning condition-specific experimental evidence on PPIs as well as its solution of integrating additional data equally apply to the study of alterations in molecular networks [14, 15]. Most biologically-motivated differential network methods, regardless whether they depict physical interactions between proteins or another kind of pairwise relation, utilize a data-type dependent correlation measure to assess rewiring [16–19]. Other methods put a stronger focus on the topology of the networks [20] or additionally make use of heterogeneous ontology information [21]. Conceptionally, correlation of gene expression is a reasonable measure of pairwise association in the context of biological interactions between genes or corresponding proteins. In the very case of protein interactions, however, the notion does neither unveil which transcriptomic alteration caused a rewiring nor provide sufficient information to assess the implications of alternative splicing (AS) events. Although AS has a substantial effect on the wiring of the interactome [22–25], it is not yet accounted for by any current computational approach.

We recently introduced PPIXpress [26], a PPIN contextualization method that enables users to account for the effect of AS events on the interactome based on transcript-level expression data. Using knowledge on the viable interactions between protein domains and the domain composition of protein isoforms, the method first relates each protein interaction in the full PPIN to an underlying domain interaction. Then it uses this correspondence to infer the condition-specific presence of PPIs given the protein isoforms indicated by the expression data. Non-transcriptomic effects on protein interactions are not covered by this approach. As an extension of this work, we propose here the differential PPIN tool PPICompare that compares the inferred interactomes between samples of two groups and tracks the cause of each alteration. The tool determines statistically significant between-group rewiring events and annotates each rewiring process with the underlying cause (one or both corresponding genes missing, or interacting domains missing due to differential transcript usage). Also, PPICompare constructs a small set of the most relevant alterations to the transcriptome that explain all systematic differences in the networks. A first application of the novel software is presented on the example of hematopoiesis [27] using data generated by the BLUEPRINT epigenome project [28–30]. To our best knowledge this work represents the first study of rewiring processes of the protein interactome during development with similar scope and granularity.

## Methods

### PPICompare

PPICompare is currently designed to be used as an extension to our tool PPIXpress for constructing condition-specific protein interaction networks [26] but can also be applied to suitable input data generated in alternative ways. As basis for the subsequent analysis, contextualized PPINs are constructed with PPIXpress for each transcript expression sample. This is explained in detail in the subsection “Constructing blood cell interactomes” below.

Given two groups of condition-specific PPINs built from the same reference PPIN, PPICompare detects all interactions that are significantly rewired between samples of the groups. In [26] we presented the underlying principle of the statistical model and applied it to the special type of matched datasets in a case study on breast cancer. Here, we extended the methodology to arbitrary groups of networks and provide a stand-alone software tool for this type of analysis. All output is written to files in the format of node- and edge-attribute tables that can be imported into other tools like, for example, Cytoscape [31]. A platform-independent Java 8 implementation of PPICompare that is able to efficiently utilize current multi-core CPUs is freely available at https://sourceforge.net/projects/ppicompare/. A user guide and example data are provided together with a precompiled executable and the complete source code.

For both practical as well as biological reasons discussed in [26], PPIXpress only adjusts the presence of interactions according to the expression data but does not alter their weight annotations. Consequently, a differential analysis of the derived networks is done based on discretized information. While discretization always implies a loss of information, it also simplifies the state space of the problem considerably and it can deflate noisy data. Advantages and disadvantages of using discretized expression data are discussed in [32], for example.

**Fig. 1:**
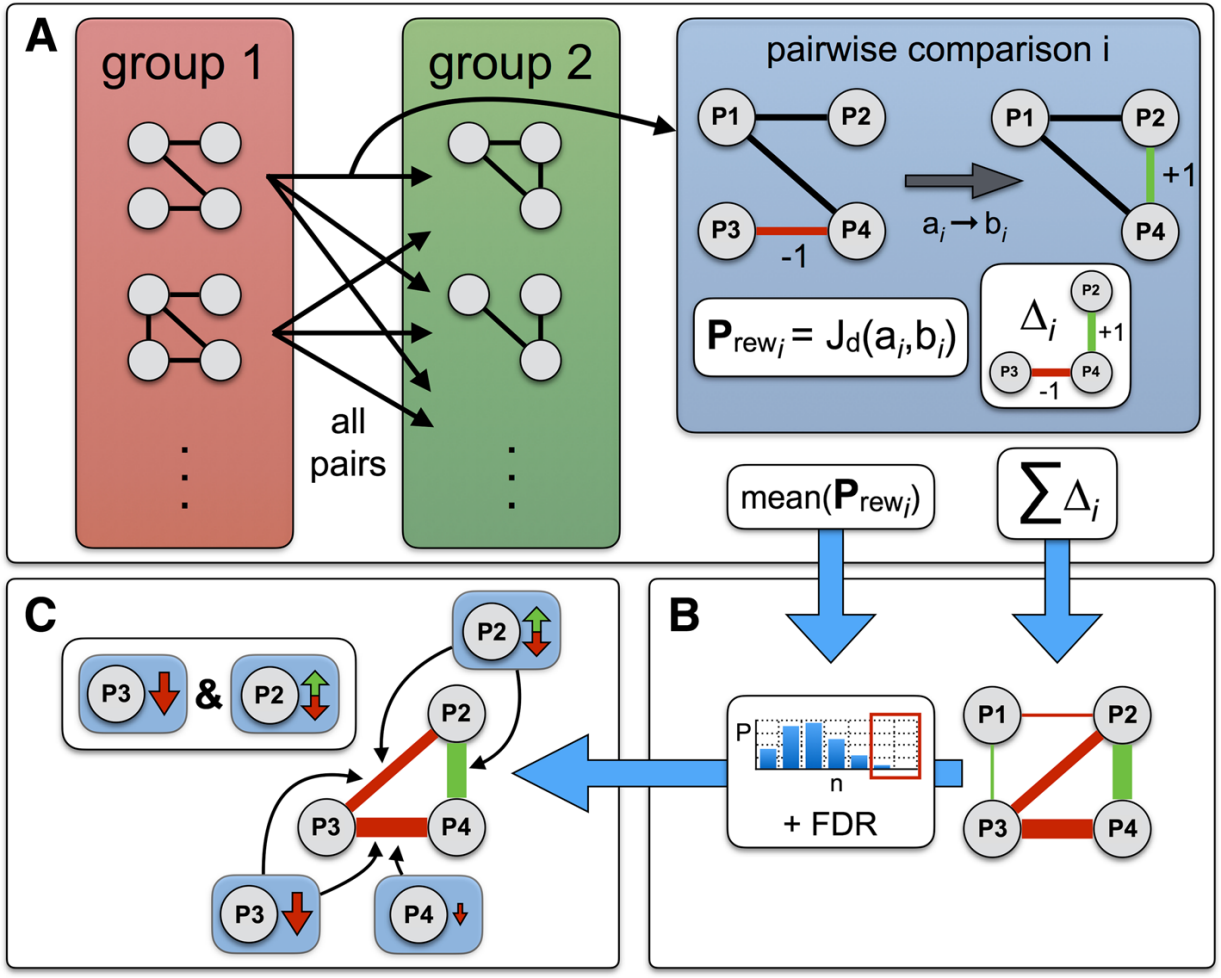
Workflow of PPICompare. **a** Examine the interactome differences between all inter-group pairs of samples. **b** Assess the significance of and the reasons for each rewiring event. **c** Discern a small set of likely changes in the transcriptome that explain the rewiring. Details are described in the main text

Figure 1 outlines the individual steps in the workflow of PPICompare. The details of panels A) to C) are described in the following three paragraphs.

#### A) Examining the interactome differences between all inter-group pairs of samples

In the first stage of the differential analysis (Fig. 1 a), each sample in the first group is compared to each sample in the second group in terms of their PPIs. Ideally, a group of samples stands here for a representative distribution of interactomes for a condition under study. For every pairwise comparison *i* a differential network *Δ*_*i*_ monitors whether a particular interaction (*u*,*v*) between proteins *u* and *v* is only found in one of the two groups. PPICompare considers the first group as the reference system and the second group is compared to it. An interaction (*u*,*v*) that is exclusively found in the sample of the second group is thus noted as *Δ*_*i*_(*u*,*v*)=+1. Likewise, an interaction (*u*,*v*) lost in the second group is noted as *Δ*_*i*_(*u*,*v*)=−1. All *N* individual pairwise observations are weighted equally and summed up to obtain a global differential network *Δ* whereby each edge (interaction) is annotated with the signed number of changes affecting it in the inter-group comparisons: $\Delta (u,v)=\sum _{i}^{N} \Delta _{i}(u,v)$. As a result of this, rewiring events with opposing observations, where both addition and removal events were detected for the same interaction, are downweighted in a natural way. The unchanged portion of the interactome does not appear at all in the differential network. Potentially emerging null-sum annotated edges in the cumulative network *Δ* are removed after the summarization.

Besides tracking the amount of rewiring per edge, PPICompare quantifies the fraction of interactions that are changed in each pairwise comparison *i* by a rewiring probability $\phantom {\dot {i}\!}\mathrm {P}_{\text {rew}_{i}}$. We defined $\phantom {\dot {i}\!}\mathrm {P}_{\text {rew}_{i}}$ as the number of rewired interactions normalized by the size of the union of interactions in both samples. This is basically the Jaccard distance [33] of the edge set. Thus $\phantom {\dot {i}\!}\mathrm {P}_{\text {rew}_{i}} = 1 - \frac {|a_{i} \cap b_{i}|}{|a_{i} \cup b_{i}|}$, where *a*_*i*_ and *b*_*i*_ are the respective sets of interactions in the samples compared in comparison *i*. In the matched comparison scheme of [26] we used the number of interactions of the smaller one of both PPINs as a stringent normalization factor. Taking here the union of the corresponding interaction sets for normalization in the Jaccard distance allows application of the method to more variable non-matched data, because a value in [0,1] is ensured. Note that all pairwise comparisons are independent from each other. The final inter-group rewiring probability P_rew_ is then obtained as the average of all individual pairwise probabilities $\phantom {\dot {i}\!}\mathrm {P}_{\text {rew}_{i}}: \mathrm {P}_{\text {rew}} = \frac {1}{N} \sum _{i}^{N} \mathrm {P}_{\text {rew}_{i}}$.

#### B) Assessing the significance of and the reasons for each rewiring event

*P*_rew_ can be interpreted as the probability of each interaction to be rewired. A one-tailed binomial test is then used to assess the statistical significance of candidate rewiring events (*u*,*v*) in the differential network *Δ* against this background (Fig. 1 b). For each candidate (*u*,*v*)∈*Δ* and a given P_rew_, PPICompare computes the likeliness of observing at least the annotated number of rewiring events |*Δ*(*u*,*v*)| over all *N* pairwise comparisons by chance:

$$p_{(u,v)} = 1 - \sum\limits_{i=0}^{|\Delta(u,v)|-1} {N \choose i} (P_{\text{rew}})^{i} (1-P_{\text{rew}})^{N-i} \text{.} $$

The *p*-values are subsequently adjusted using the Benjamini-Hochberg procedure [34]. Only rewiring events below a user-defined false discovery rate (FDR) threshold are processed further and reported.

Since version 1.05, PPIXpress can optionally report the major isoform that was associated with each individual protein during the construction of the condition-specific interaction network. As a consequence, PPICompare can use the output of PPIXpress to exactly reproduce and annotate which change or which changes in the transcriptome altered an interaction between samples of the two groups. Since each interaction depends on the presence as well as the compatibility of both interacting proteins, the two essential causes of rewiring events are either a major shift in the abundance of at least one interaction partner between groups (differential expression, DE), or a switch of the major isoform of at least one of the proteins that alters the domain composition in a way that affects the interactome (alternative splicing, AS). Whereas alterations to both proteins are in principle not neccessary to explain changes to an interaction, even redundant pairs of causes are explicitly monitored by PPICompare because they could point to a different mode of regulation, such as the purposeful coexpression of complex partners. PPICompare determines and reports the individual distributions of all causal reasons for each significantly rewired interaction.

#### C) Discerning a small set of likely changes in the transcriptome that explain the rewiring

To identify the events that caused the systematic rewiring of the PPINs between the groups under study, it is reasonable to look for a set of transcriptomic changes that is both very likely given the data and of small cardinality.

The association of causes and affected interactions can be thought of as a bipartite graph, where one class of nodes are the significantly rewired interactions and the second class are individual causal reasons (change in expression or splice form of a single protein). In such a graph, the alterations point to the interactions they affect (see Fig. 1 c). Here, we tracked how often a transcriptomic cause *i* is relevant for each rewiring event. Thus, we know the number of pairwise comparisons *p* *w*_*i*_ in which the alteration happened and the number of significantly rewired interactions *r* *w*_*i*_ that were affected by it. Since the importance of a rewiring reason *i* should be related to its frequency across all comparisons and rewired interactions, we score each one with *s*_*i*_=*p* *w*_*i*_×*r* *w*_*i*_. Determining then a small set of those reasons that explain all rewiring events and consists of preferably important members is a weighted set-cover problem [35].

As this problem is classically defined as a minimization problem, we converted the scores *s*_*i*_ into weights *w*_*i*_ by setting *w*_*i*_=*s*_*max*_−*s*_*i*_, where *s*_*max*_= max(*s*_*i*_)+1. The addition of one prevents the possibility of numerical equality and subsequent loss of information in the ratio that is then optimized. To efficiently solve this weighted set-cover problem for large instances, PPICompare implements a greedy algorithm with provable performance guarantees [36]. The algorithm repeatedly selects the rewiring reason *i* with the minimum ratio of *w*_*i*_ divided by the number of rewiring events that it additionally explains. This is done until all significant rewiring events are covered. The resulting solution set is part of the standard output of PPICompare.

Note that the notion of a reduced set refers here to the relevance in the interaction networks only. At a higher level, some crucial alteration which is not necessarily of transcriptomic origin and is simply not reflected in the differential interactome may, of course, reside upstream in the hierarchy of causal regulatory effects and thus be of more importance.

### Constructing blood cell interactomes

Specific PPINs for samples of 11 hematopoietic cell types were constructed on the basis of transcript expression data from the 7 ^th^ data release (Sept. 2015) of the BLUEPRINT epigenome project [28–30]. From the provided preprocessed data of the consortium we considered all samples of blood stem cells and precursors derived from cord blood and all samples of common mature cell types derived from venous blood that had at least 3 samples for this tissue of origin. The downloaded data included RNA-seq data on hematopoetic stem cells (HSCs, 6 samples), multipotent progenitors (MPPs, 3 samples), common myeloid progenitors (CMPs, 3 samples), common lymphoid progenitors (CLPs, 5 samples), megakaryocyte erythrocyte progenitors (MEPs, 4 samples), granulocyte monocyte progenitors (GMPs, 3 samples), erythroblasts (EBs, 7 samples), and megakaryocytes (MKs, 5 samples). Regarding common mature cell types that met those criteria we obtained data for neutrophils (Ns, 10 samples), monocytes (Ms, 5 samples), and naive CD4 T cells (CD4s, 8 samples).

**Fig. 2:**
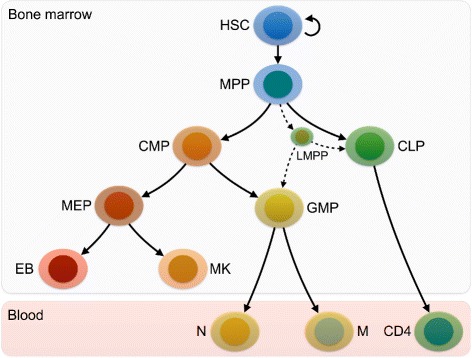
Hierarchy of hematopoietic differentiation stages used as basis for our study. For reasons discussed in the main text, we only considered classical ontological relationships for all analyses (*solid lines*) and did not include more recent models and their accompanying novel entities. Lymphomyeloid-restricted progenitors (LMPPs, first proposed by [44]) are shown as an example for emerging relationships that are not covered by our data (*dotted lines*). In this model, MPP, CMP, MEP and GMP are developmental branching points and will be investigated in detail later

For consistency, we followed the strategy used in [30] from which we took our input data and of others who investigated blood cell types during development [37–39]. Thus, we based our analyses on the ontological relationships defined by the classical dichotomy model of hematopoiesis [27, 40]. Although recent insights based on data from single-cell sequencing challenge this established model of hematopoiesis, the model characterized by the BLUEPRINT data was not analyzed with respect to protein interactions so far and there appears to be no clear consensus on a revised model yet [41–45]. Figure 2 shows a schematic representation of the developmental relationships among the cell types we examined.

The preprocessed RNA-seq data of the 7 ^th^ BLUEPRINT release was quantified with RSEM [46]. For better comparability between samples [47, 48], PPIXpress uses transcripts per million (TPM) as the relevant expression measure for RSEM output files. For all transcript expression samples we built protein interaction networks with PPIXpress (version 1.08) for a range of TPM thresholds from 0.0 TPM to 1.0 TPM in steps of 0.01. This means that only proteins with an associated transcript that was expressed above this cutoff were considered in the respective network contextualizations. Using PPIXpress, we retrieved the full protein interaction network for human (taxon 9606) from mentha [49] (data of 18. Jan 2016). Outdated Uniprot accession numbers (release 2015_12) [50] were updated automatically by PPIXpress. The resulting human reference protein interaction network contained information on 221,158 physical interactions between 17,292 proteins. Furthermore, PPIXpress retrieved annotation data from Ensembl (release 83) [51] and domain interaction data from 3did (release July 2015) [52] and iPfam (version 1.0) [53] for the mapping of protein interactions to domain interactions. With this data, 49.1% of the proteins in the reference PPIN were annotated with at least one domain that contributed to the PPI association. 20.3% of the PPIs were covered by domain interactions and thus may be potentially altered by AS events that our model can capture. Note that this partial coverage is in an expected range for domain annotations and domain-domain interaction data [26]. Interations that are mediated by disordered regions between such conserved domains are currently not considered by PPIXpress. These practical limitations certainly confine the ability of the pipeline to detect the contribution of AS on the in-vivo rewiring of the proteome in its entirety. See [26] for more details concerning the methodology.

To establish a good TPM threshold, we utilized additional independent data on proteome abundance from the Human Proteome Map (HPM) [54] on individual hematopoietic cell types. We used their mass-spectrometry data on the abundance of proteins mapped to HGNC protein-coding genes [55] and considered each protein as present if its corresponding abundance value was larger than zero.

### Data evaluation

#### Participation in complexes, annotational homogeneity, and betweenness of interactions

To determine whether an interaction within a known complex is rewired, we downloaded the data on human protein complexes from CORUM (release Feb. 2012) [56] and checked whether interacting protein pairs belong to a known complex.

Furthermore, we annotated all interactions in our reference PPIN with the semantic similarity of the interactors concerning the three GO ontologies biological process (BP), molecular function (MF), and cellular compartment (CC) [57]. Semantic similarities were obtained using GOSemSim (version 1.28.2) [58] with default options and annotation data from org.Hs.eg.db (version 3.2.3) [59]. Also, we determined the betweenness of the interactions, which is the normalized sum of the fraction of all-pairs shortest paths that include this interaction. Betweenness values were computed with NetworkX (version 1.10) [60] on the basis of the reference PPIN.

#### Association and enrichment of rewiring events within pathways

We mapped deregulated interactions to the biological pathways they might affect. A related approach based on the coexpression between adjacent genes in pathways was proposed by [16] and termed Edge Set Enrichment Analysis (ESEA).

We retrieved preprocessed KEGG [61] and Reactome [62] pathway data as undirected graphs from the ESEA R package (version 1.0) [16] and converted the HGNC gene names to UniProt accessions using mapping data from the HGNC webservice (accessed on March 26th, 2016) [55]. We followed the example of [16] and only considered pathways with at least 15 and at most 1,000 connections in the original pathway data. The remaining pathway-annotated links were then related to the exact interactions in our reference interactome data. 3,394 PPIs (1.5% of our reference PPIN) among 1,624 proteins (9.4% of our reference PPIN) could be exactly mapped to 106 KEGG pathways. 7,318 PPIs (3.3% of our reference PPIN) among 2,617 proteins (15.1% of our reference PPIN) corresponded to 495 Reactome pathways. Enrichment of pathways was calculated on the basis of a hypergeometric test as is often done for gene sets [63]. *P*-values were subsequently adjusted for KEGG and Reactome pathways independently using the Benjamini-Hochberg procedure [34]. Since PPICompare only distinguishes between rewiring events that are statistically significant and those that are not, the GSEA-based approach [64] of ESEA to identify pathway enrichment is not applicable for our task.

#### Unspecific enrichment analysis of deregulated proteins using DAVID

Unspecific protein-set enrichment analysis was conducted with the DAVID webservice (version 6.7) [65] using default settings. We set all proteins in the reference PPIN as the background for the analysis. The reported significances of term enrichments refer to the *p*-values adjusted using the Benjamini-Hochberg correction [34].

#### Proteins relevant to hematopoiesis and their regulatory targets

As proteins relevant to blood development, we considered all human proteins annotated with GO term GO:0030097 (“hemopoiesis”) using QuickGO [66] on May 30th, 2016. In our reference PPIN this was the case for 480 proteins. We refer to these as “hematopoiesis proteins” in the remaining text. Additionally, we downloaded literature-curated annotations of experimentally validated gene regulatory relationships in human from the TRRUST database (version 12/08/2014) [67]. The regulatory network contained data on 727 transcription factors and 7906 interactions between proteins in the reference interactome. Among these transcription factors were 101 hematopoiesis proteins. Combining both data sources, 1274 proteins were either hematopoiesis proteins or proteins directly regulated by a hematopoietic transcription factor.

Enrichment of a query regarding a specific protein set defined by this data was then determined using a hypergeometric test. As protein sets we analysed the combined set of hematopoiesis proteins and targets of hematopoietic transcription factors, the set of hematopoiesis proteins, and its subset of hematopoietic transcription factors.

Furthermore, we determined enrichment of targets associated with transcription factors covered by our regulatory data. Since the sets of targets of each transcription factor were tested individually, the *p*-values for each transcription factor were subsequently adjusted using the Benjamini-Hochberg procedure [34].

## Results and discussion

Using PPIXpress and transcript expression data from BLUEPRINT we constructed the protein interactomes of 59 samples representing 11 different types of blood cells for different expression thresholds (see “Methods” section). To ensure that the biological analyses regarding developmental transitions were based on a single expression discretization parameter that best reflects the actual protein concentrations in the cell, we used mass spectrometry-based proteome abundance data from HPM [54] for guidance (see Additional file 1: Results S1.1). All further analyses presented were performed on the protein interaction networks constructed with the HPM-derived threshold of 0.31 TPM. Furthermore, we checked by a subsampling approach how robust the rewiring detection methodology was if only a small number of samples was available for comparison (see Additional file 1: Results S1.2). Apparently, groups with at least 3 samples provide meaningful results. As there is no computational pipeline with comparable features and scope, we did not contrast PPICompare with other tools.

### The rewiring of the blood interactome during development

For a biological interpretation of the derived protein interaction networks, we compared all cell types that are adjacent in developmental progression according to the classical model of hematopoiesis as depicted in Fig. 2. PPICompare (version 1.0) was applied to the corresponding PPINs generated with HPM-optimal threshold and the default FDR of 0.05. Table 1 summarizes the differences in the interactome sizes detected at developmental transitions.

**Table 1:** Quantitative changes of blood interactomes during developmental transitions

	Protein interaction networks		PPICompare results			
Transition	Interactome sizes n_i_→n_j_	*Δ*n_i_→n_j_	P_rew_	obs_s_/obs_all_	rew_+_/rew_−_	rew_+_−rew_−_
HSC →MPP	101,235 ± 30,315→111,556 ± 10,069	10,321 ± 31,944	0.372	15/18 (0.83)	311/123	188 (0.32 *σ*)
MPP →CMP	111,556 ± 10,069→117,254 ± 3176	5698 ± 10,558	0.278	9/9 (1.00)	856/423	433 (0.50 *σ*)
MPP →CLP	111,556 ± 10,069→79,383 ± 31,849	–32,173 ± 33,402	0.455	15/15 (1.00)	1/705	–704 (0.94 *σ*)
CMP →MEP	117,254 ± 3176→117,768 ± 8692	513 ± 9254	0.261	8/12 (0.67)	3955/2532	1423 (0.10 *σ*)
CMP →GMP	117,254 ± 3176→121,051 ± 6427	3796 ± 7169	0.256	6/9 (0.67)	8468/5556	2912 (0.12 *σ*)
MEP →EB	117,768 ± 8692→111,326 ± 28,549	–6441 ± 29,842	0.348	20/28 (0.71)	3021/4146	–1125 (0.18 *σ*)
MEP →MK	117,768 ± 8692→132,598 ± 8456	14,831 ± 12,126	0.293	12/20 (0.60)	10,574/3848	6726 (0.67 *σ*)
GMP →N	121,051 ± 6427→67,007 ± 9203	–54,044 ± 11,225	0.585	24/30 (0.80)	3895/41,599	–37,704 (1.46 *σ*)
GMP →M	121,051 ± 6427→113,534 ± 2762	–7517 ± 6995	0.337	10/15 (0.67)	15,763/21,407	–5644 (0.27 *σ*)
CLP →CD4	79,383 ± 31,849→120,282 ± 19,498	40,898 ± 37,343	0.512	30/40 (0.75)	17,181/1919	15,262 (0.69 *σ*)

The net change in number of interactions *Δ* *n*_*i*_→*n*_*j*_ is reported as the mean difference between all samples per cell type and its standard deviation. obs_s_ is the minimum number of rewired obervations out of all pairwise comparisons obs_all_ that were necessary for a rewiring event to be called significant in PPICompare applied to that transition. For increased comparability, the fraction as a floating-point number is given in brackets. The number of rewiring events deemed significant by PPICompare is depicted as rew_+_ for emerging interactions and rew_-_ for vanishing interactions. In addition to the net change among significant rewiring events, its absolute deviation to *Δ* *n*_*i*_→*n*_*j*_ in terms of standard deviations *σ*(*Δ* *n*_*i*_→*n*_*j*_) is shown in brackets

#### Developmental branching points associated with lineage commitment are most distinct in terms of quantitative rewiring

Without a tool such as PPICompare, the average net difference in the number of interactions between proteins *Δ* *n*_*i*_→*n*_*j*_ (third column) is the only differential measure that can be analyzed. On its own, it provides little information on how many and no information on which interactions actually emerged or vanished during a conversion from *i* to *j*. For two of the four developmental branching points that were considered in our model of blood development (see Fig. 2), the net difference even had a different sign depending on the direction of the transition in the branch. Interestingly, this was exactly the case when a bifurcation is passed that determines a lineage choice, namely, when descendant cells of MPPs either evolve toward the erythro-myeloid (MPP →CMP) or toward the lymphoid lineage (MPP →CLP) and, later in the developmental tree, when descandants of MEPs belong either to the erythroid (MEP →EB) or to the myeloid lineage (MEP →MK).

**Fig. 3:**
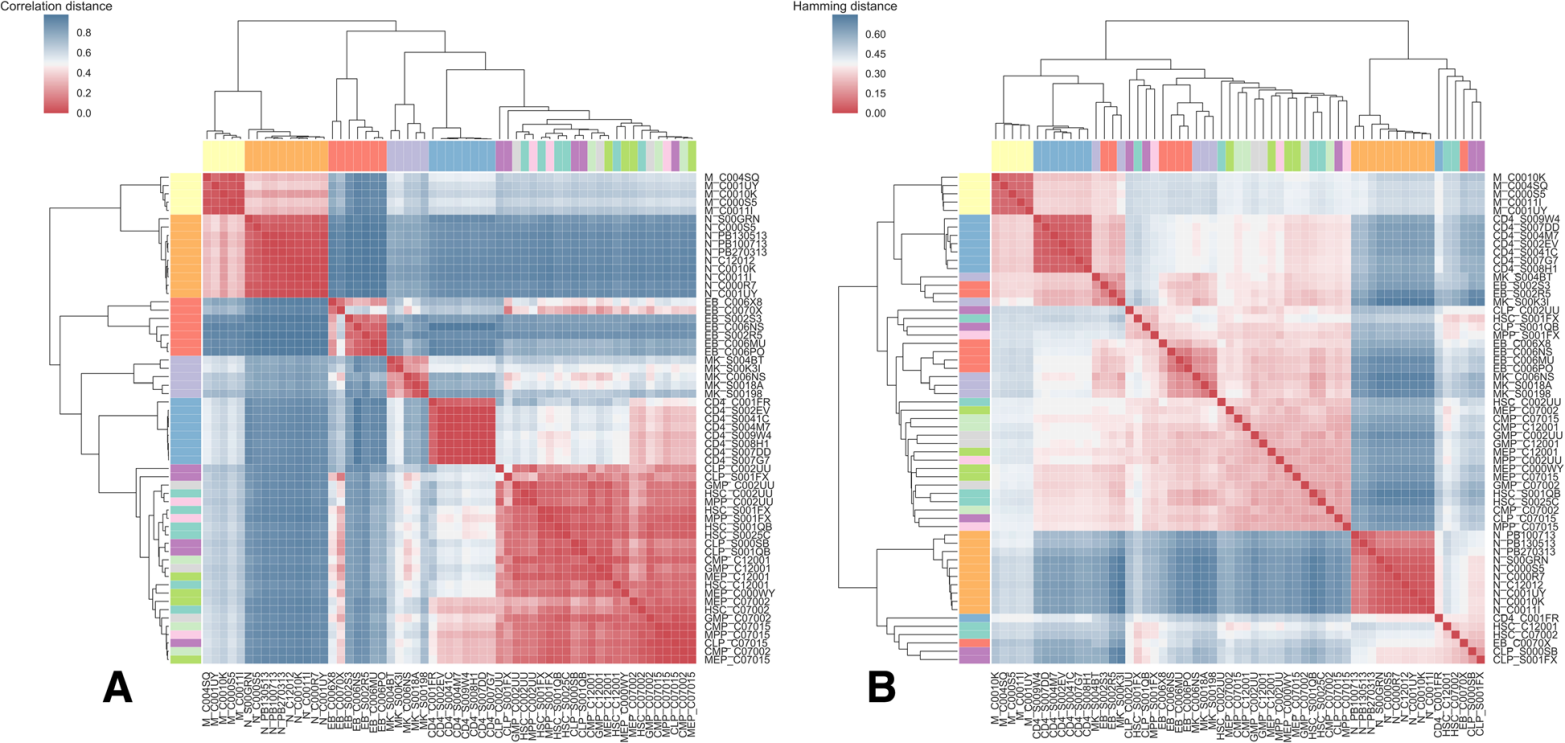
Hierarchical clustering of hematopoiesis cell types. Results of average linkage clustering (UPGMA) applied to all samples based on **a** the correlation of the transcript expression data (vector of expression values for transcripts associated with a Uniprot accession in Ensembl 83) and **b** the normalized Hamming distance between inferred protein interactomes (boolean vector of abundance concerning all significantly rewired interactions). Cell types are additionally distinguished by *colored labels*

As a consequence of the high variance among the network sizes of most cell types, the standard deviation *σ*(*Δ* *n*_*i*_→*n*_*j*_) was larger than its mean change for most developmental steps. We analyzed whether this within-group variance is an artefact from the network discretization. Yet, the interactome sizes showed a similar variability when all transcripts with non-zero expression (equivalent to a TPM threshold of 0.0) were presumed abundant for each cell type instead of the stricter threshold used in the analyses (see Additional file 2: Table S1). Furthermore, hierarchical clustering of the original expression data was not able to distinguish the progenitor cell types properly (see Fig. 3 a). Thus, the high variability seems inherent to the data. Besides, clustering on the basis of the inferred interactomes had problems to properly separate some other cell types (see Fig. 3 b) which were also grouped suboptimally when clustered by discretized expression data (see Additional file 1: Figure S1). Heterogeneity is common in this context because cell populations that were separated by specific surface markers often still contain hidden diversity in the form of subpopulations. Sample variability, but also the dilution of it, is therefore a general issue for averaged snapshots made in bulk measurements of such samples [68, 69]. A high degree of transcriptomic heterogeneity within grouped cell types of the hematopoietic system is well-described for early developmental stages [42, 43, 45, 70] and also for various terminal cell types [71–73].

#### PPICompare reports a reasonable amount of rewiring events

With PPICompare we identified for all developmental steps the statistically significant subsets of emerging (rew_+_) and vanishing (rew_-_) interactions. From this, the net change rew_+_−rew_-_ was computed. The direction of this net change of detected interactions was always the same as that of the observable mean net difference although this must not necessarily be the case. With the exception of the transition CMP →MEP, the absolute change according to rew_+_−rew_-_ was always smaller than *Δ* *n*_*i*_→*n*_*j*_. Considering that the tool requires relevant rewiring events to occur sufficiently more often than expected from the rewiring background, it is not surprising that it provided smaller absolute estimates. Still, the deviation of the PPIXpress estimate from the mean net rewired interactions was within 0.5*σ* for most transitions and well below 1.5*σ* for all transitions we examined. Furthermore, *P*_rew_ and *σ*(*Δ* *n*_*i*_→*n*_*j*_) were positively correlated (Pearson corr. coeff. 0.82). The statistical criterion used to filter out the significant portion of the differential interactome ensures to withdraw all rewiring events of questionable relevance. If one aims at also uncovering slight alterations, PPICompare is best applied to grouped samples that deviate as little as possible between groups.

Adding to that, the magnitude of the absolute net change hides the actual amount of rewiring. In the developmental transition GMP →M, for example, the 37,170 rewired interactions (17% of the complete interactome known in human) considered significant by PPICompare only entailed an absolute net change of 5644 interactions. As a side note, neither obs_s_/obs_all_ and *P*_rew_ (Pearson corr. coeff. 0.3) nor obs_s_/obs_all_ and obs_all_ (Pearson corr. coeff. -0.15) were correlated and PPICompare determined a wide range of significance thresholds from 60% of all observations up to all comparisons for individual transitions. This shows that the statistical model adapted to the individual set of between-group rewired interactions independent of the rewiring probability and the number of samples.

Unfortunately there is neither a gold-standard nor a representative set of qualitative statements for comparison. For the non-terminal developmental stages in human bone marrow (all but the lower 3 rows) the very first transition HSC →MPP was reported to be mostly driven by the deregulation of non-protein-coding transcripts, whereas protein-coding transcripts were more important in later stages [30]. Furthermore, quantitative proteome and transcriptome analyses of mouse HSC and MPP populations likewise showed that protein abundance and transcript levels were correlated positively and few proteins were differentially expressed (47 of 4037 assessed proteins) [74]. If those findings are transferred to the interactome, fewer changes should be expected in the transition at the apex of the hierarchy than in later transitions. This was indeed the case for the results of PPICompare but less so for the mean net difference.

### A causal view on the rewiring of the blood interactome during development

**Fig. 4:**
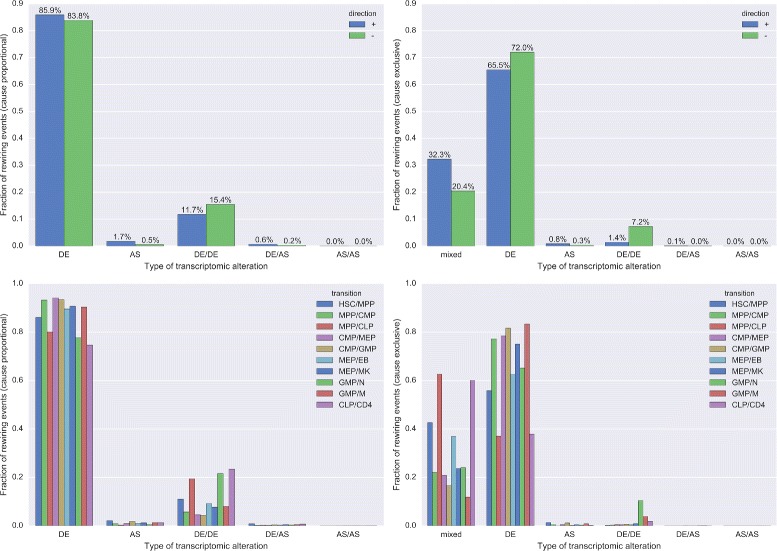
Distribution of the transcriptomic alterations that entailed significant rewiring events. Shown is the impact of the considered types of expression changes on interaction partners regarding all individual rewiring events per transition. The types were weighted by their proportional contribution to each event during the pairwise comparison step (*left plots*) or as the sole contributing cause (*right plots*). In the latter case, rewiring events that had more than one explanatory transcriptomic cause in a transition were annotated as “mixed”. The types of causes were either normalized by the direction of the rewiring events (*upper plots*) or by their contributions to individual transitions (*lower plots*). In the *top plots*, “+” (*blue*) means emerging interactions and “-” (*green*) means vanishing interactions. The bottom three developmental transitions are those towards terminally differentiated cell types found in blood (GMP/N, GMP/M, and CLP/CD4)

Next we examined which changes to the transcriptome caused interactions to emerge or vanish when direct developmental descendants were compared. For each significantly rewired interaction, PPICompare automatically tracks how often transcriptomic alterations of the interactors occur during the pairwise comparison between groups. The causal deregulation events that are covered by the method can be classified either as differential expression of one of the two genes coding for the interaction partners (DE), alternative splicing of one partner (AS), or corresponding transcriptomic changes to both partners (DE/DE, DE/AS and AS/AS). We analyzed in two different ways how these modes of PPI-regulation contributed to the differential interactome during hematopoiesis. First, since more than one type of transcriptomic alteration may have been detected, we weighted the contribution of each type proportionally to its occurrence in each rewired interaction (cause proportional). Secondly, we only allowed a single type per rewired interaction and else classified its causing type as ”mixed” (cause exclusive). Table 2 lists aggregated results over all state transitions. Figure 4 provides details for individual transitions.

**Table 2:** Distribution of the transcriptomic alterations that entailed significant rewiring events

Type	Cause proportional [%]	Cause exclusive [%]	Absolute loss [%]	Relative loss [%]
DE	84.73	69.14	15.59	18.40
AS	1.03	0.53	0.49	48.06
DE/DE	13.81	4.67	9.14	66.19
DE/AS	0.41	0.06	0.36	85.81
AS/AS	0.02	0.00	0.01	87.23
mixed	0.00	25.60	/	/

Shown is the impact of conceivable types of expression changes on interaction partners regarding all individual rewiring events per transition. The six types of expression changes were weighted by their proportional contribution to each event during the pairwise comparison step (cause proportional) or as the sole contributing cause (cause exclusive). In the latter case, rewiring events that had more than one explanatory transcriptomic cause in a transition were annotated as “mixed”. Additionally, the amount of causal relevance lost due to this stricter notion is given as percentage in absolute and relative terms

#### Differential gene expression of a single protein is the prevalent cause of rewiring for developmentally sequential adjacent blood cell types

Overall and for both types of analyses, most statistically significant changes to the interactome during hematopoiesis were driven by differential expression of a single protein, followed by differential expression of both partners, and by AS of a single one. The combinations of differential expression and AS of one partner each and AS of both interacting proteins were only relevant in few cases (see Additional file 3: Table S2). Imbalance concerning the direction of changes for individual modes of deregulation (see upper panels of Fig. 4) was mostly caused by the considerable share of individual transitions to all rewiring events. More than half of the “mixed” events describing emerging interactions can be attributed to the strongly net positive change of the transition CLP →CD4. An even larger fraction of the vanishing “mixed" events and more than three quarters of the vanishing DE/DE events stem from GMP →N (see Additional file 3: Table S2). Rewiring events solely driven by AS ocurred more frequently in emerging interactions. This directional bias was independent of the net change of all contributing transitions (see Additional file 3: Table S2). We noted no preference of rewiring events driven by AS of one interaction partner towards either early or late developmental stages (see lower panels of Fig. 4).

This general order of importance that we observed for the different modes of deregulation, in particular DE being more prevalent than AS, seems plausible. We already mentioned possibly confounding factors such as the incomplete coverage of the interactome with domain annotation data that PPIXpress uses to detect AS events of influence (only about half of the proteins and a fifth of the interactions in the reference interactome are covered, see “Methods” section). Despite of this missing information, regulation of gene expression is generally considered to be the main determinant of cellular specificity [75, 76] whereas splicing is more relevant between individuals [77]. The contribution of AS, however, certainly depends on the developmental system under study and is likely to be higher in the human brain [78, 79], for example.

#### Alternative splicing is necessary to explain many significant rewiring events in hematopoiesis

Although the contribution of AS seems minor in comparison to differential gene expression (below 1% in exclusive causes), 871 rewiring events across all developmental transitions considered here could only be fully explained by including AS (see AS, DE/AS and AS/AS in Additional file 3: Table S2). These cases would have been missed by methods that only rely on gene expression. Rewiring events that were exclusively regulated by AS across all comparisons in a transition were enriched (adj.*p*<0.05) in pathway annotations concerned with the post-elongation processing of mRNA (affecting genes associated with splicing and polyadenylation), the cell cycle (G2-M checkpoint and control of the pre-replication complex by the activator of S phase kinase DBF4), transcription initiation, the transport of mRNA, as well as the regulation of phagocytosis (see Additional file 4: Table S3 for details on interactions, databases and pathway terms). Our approach to determine interaction-centric enrichment of pathway annotations is outlined in the “Methods” section.

For example, we found that three genes which code for components of the spliceosome complex (PRPF4B, SNRNP70, SRSF3) switched their major isoform to a variant that undergoes nonsense-mediated decay (NMD) at specific points during blood development and therefore did not produce functional protein products anymore. This regulatory mechanism has been described for several splicing factors like SRSF3 (Serine/arginine-rich splicing factor 3) [80, 81], which we found to be turned off during the transitions GMP →N and CLP →CD4. We found that this was also the case for SNRNP70 (U1 small nuclear ribonucleoprotein 70 kDa) in the transition CMP →GMP. The protein was then activated again in the GMP →M transition but not in the branching to neutrophils (where SRSF3 was also deactivated). In [76], spliced protein isoforms detectable in mass spectrometry were also enriched with nuclear ribonucleoproteins. Furthermore, PRPF4B (Serine/threonine-protein kinase PRP4 homolog) switched to an active isoform in GMP →M. Since PPIXpress (version 1.08) only uses domain annotations of protein-coding transcripts, protein interactions that were associated with a domain interaction were correctly predicted to vanish if the corresponding transcript was classified to undergo NMD. Additional file 5: Table S4 provides a detailed listing of rewiring events associated with known protein complexes across all stages of hematopoiesis.

#### Different types of alterations can cause the same rewiring event

When we required each rewiring event to be consistently deregulated in the same way in all between-group comparisons for the respective transition, the contributions of most alteration types decreased severely by up to 87% compared to their proportional contribution. The reason for this is that they mostly occurred together with other transcriptomic changes (see last two columns of Table 2). To associate rewiring events with modes of deregulation in a definitive manner, we will use this strict interpretation of regulatory changes in the remaining text. Still, considering that individuals can show a varying composition of major protein isoforms in the same cell type [77], it is plausible that different alterations to the transcriptome may drive the same net change to their interactomes.

With the exception of the transition GMP →N, events caused by a mixture of alteration types were more prevalent in transitions with higher *P*_rew_ (see lower right distribution in Fig. 4, Pearson corr. coeff. 0.90 when GMP →N was left out). The relative loss in that regard was largest for what we will call “co-deregulatory” types of regulation in the following (rewiring events caused by DE/DE, DE/AS and AS/AS events, see last two columns of Table 2). This raises the question if simultaneous deregulation of interaction partners is actually a meaningful mode of control or if the observations where this was noted were the result of concealed individual deregulation events across different intermediate stages of development.

#### Simultaneous deregulation of interaction partners shows tendency towards rewiring within functional modules

Protein interaction networks are thought to be organized in a modular fashion. Several studies, mainly concerned with highly connected (hub) proteins in yeast, showed that there are two basic types of such proteins in interaction networks. Hub proteins either operate intramodular and are coregulated with their interaction partners to work together on the same task as a cohesive unit, or they act as intermodular connectors of different functional modules and are expressed independently of their neighbors [82–85]. Whereas those essential implications of the modular structure also apply to the human interactome, the complexity there is beyond dichotomous classification [85]. Yet, interaction partners that are specifically regulated together should more likely belong to the same functional module in the PPIN. Therefore they should also be more likely involved in the same protein complexes, work in the same biological process, have similar function, and be colocalized [82, 84]. Furthermore, the betweenness, a measure from graph theory to delineate modules, should be lower for intramodular interactions than for intermodular interactions [86, 87].

We compared rewired interactions caused by deregulation of only one interaction partner with those where the expression of both interaction partners was altered and to those with mixed causes in this respect. To test their tendency to reside in functional modules, we considered the involvement of the affected interaction partners in known CORUM protein complexes [56]. Also, we analyzed the similarity of their interaction partners regarding all GO ontologies (biological process, molecular function and cellular compartment) [57], and the betweenness of the affected interaction in the reference PPIN. The results are visualized in Additional file 1: Figure S2.

We found that rewiring events caused by simultaneous deregulation affected indeed more often known protein complexes (fraction of interactions associated with reference complexes increased from 3.8 to 5.3%) and had significantly lower betweenness (median betweenness decreased by 14%, two-sided Wilcoxon rank-sum test *p*<10^−30^). Also, co-deregulated interaction partners were more likely to work on similar processes (median GO biol. process similarity increased by 2%, two-sided Wilcoxon rank-sum test *p*<0.03) and had comparable similarities of GO mol. functions and GO cellular compartments. Taken together, these soft factors support the interpretation that co-deregulated partners in the PPIN are more likely part of the same functional module.

Interestingly, rewiring events caused by DE/DE and DE/AS were predominantly (relative and absolute) found in transitions towards the terminal developmental stages (see lower right panel of Fig. 4 and Additional file 3: Table S2). Among those, vanishing interactions during the progression of GMPs to Ns and Ms were highly enriched with annotations concerning cell cycle progression (see Additional file 6: Table S5). More specifically, interactions disappeared that are important for the G2-M checkpoint and for the activation of the pre-replication complex. For the transition CLP →CD4, this was not the case for any mode of regulation. Since T cells are proliferating [88] and Ns and Ms are cell types that are generally non-proliferating [89, 90], some of these alterations of protein interactions are likely associated with cell cycle exit.

Furthermore, GMP →N was of special interest in that regard, because it showed by far the highest amount of co-deregulation (4,786 rewiring events caused by DE/DE, DE/AS or AS/AS, see Additional file 3: Table S2) and also the largest overall amount of rewiring as indicated by *P*_rew_ (see Table 1). When analyzed in detail, the transition to terminal neutrophils is a stepwise process with five intermediate stages that are, unfortunately, not resolved by the BLUEPRINT data. Within those finer-grained steps, proliferation, in fact modulated by the expression of cell-cycle proteins, steeply decreases during an early stage and is completely absent after the very next [89]. Whereas this regulatory process is thus not completely synchronous, the net effect is still correctly described by our analysis.

Besides the deactivation of the cell cycle, a surprisingly large number of co-deregulated changes to the interactome were associated with the depletion of interactions of other coherent molecular machineries, namely RNA polymerase III (Pol III) and tRNA processing (see Additional file 6: Table S5) as well as mitochondrial ribosomes (see also Additional file 5: Table S4). This latter finding matches the fact that mitochondria are very rare in Ns and not used for energy metabolism [91, 92]. In contrast to this, the (partial) depletion of Pol III has, to our best knowledge, not been explicity described in the literature. Pol III is responsible for entirely different functions in immune cells. Its inhibition restrains phagocytosis and cytokine secretion in macrophages due to its role in tRNA production [93], but it can also act as a sensor to detect foreign DNA [94]. However, its inhibition does not alter the response of Ns in that regard [95]. Owing to the short lifespan of Ns, it may simply be an economical decision of budgeting cellular resources.

#### Small set of likely transcriptomic alterations

PPICompare provides an optimization approach that suggests a small set of likely changes in the transcriptome that explain all significant rewiring events. In every transition each of these alterations to a single protein yielded between 6.6 and 17.4 rewiring events on average (average of 11.4 over all transitions). The number of all proteins affected by any significant rewiring event was on average 5.3 times larger than the number of proteins in the respective small set of changes (see Additional file 7: Table S6). From now on, we will refer to this smaller set of proteins as the “reduced set” of proteins affected by rewiring.

**Fig. 5:**
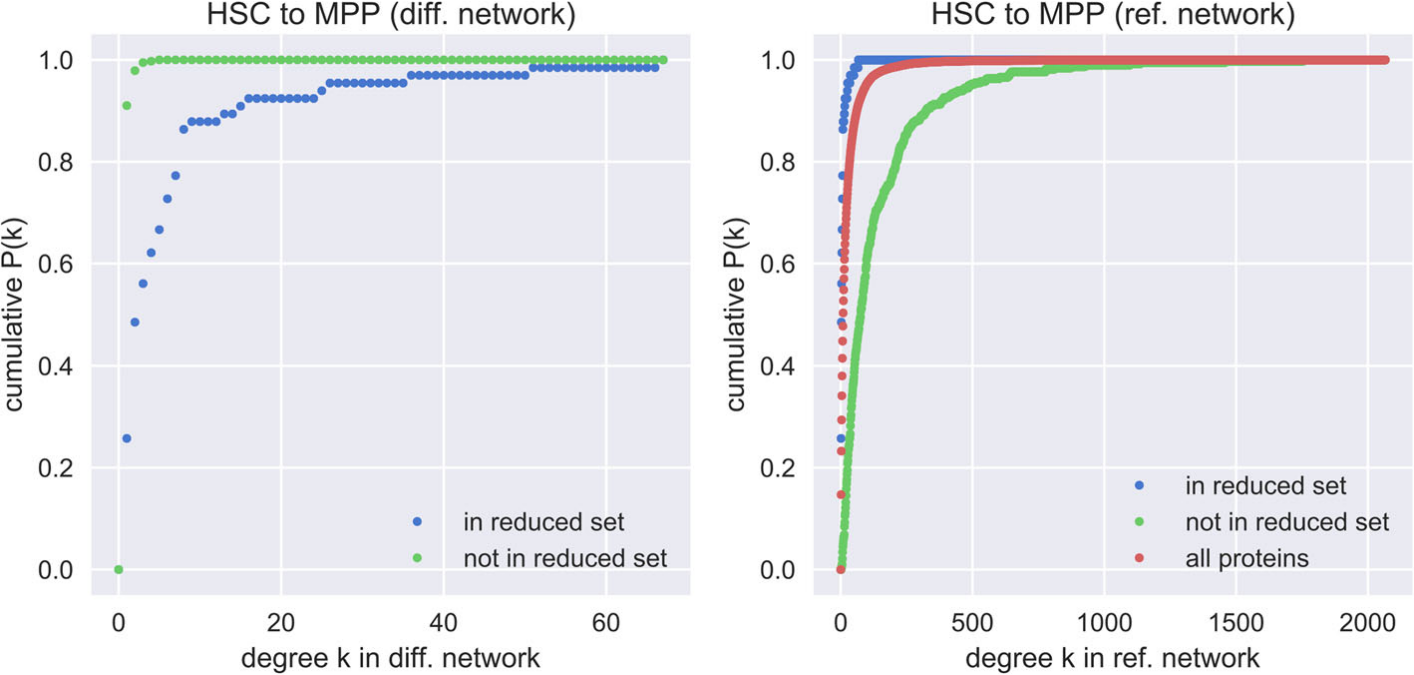
Cumulative degree distributions of rewired proteins. Cumulative degree distributions of the rewired proteins of the transition HSC →MPP in the corresponding differential network (*left*) and the distributions of the rewired proteins in the reference protein interaction network (*right*). The rewired proteins are additionally split up into those in the reduced set and the remaining ones, ”all proteins” depicts all proteins in the reference network

The optimization approach tends to select hub proteins in the differential network (see Fig. 5 (left) for an example and Additional file 1: Figure S3 (upper half) for complete results). This is not very surprising given that the score *s*_*i*_ increases if such a protein was transcriptionally deregulated. Also, it is biologically reasonable because an appropriately deregulated protein will cause rewiring around itself. Interestingly, selected proteins were not necessarily highly connected proteins in the reference interactome whereas those rewired proteins that were not in the reduced set tended to have above average degrees in the complete network (see Fig. 5 (right) for an example and Additional file 1: Figure S3 (lower half) for the complete results). The latter observation likely increased their chance of acting as interaction partner of a deregulated protein and thus be part of the differential network.

**Fig. 6:**
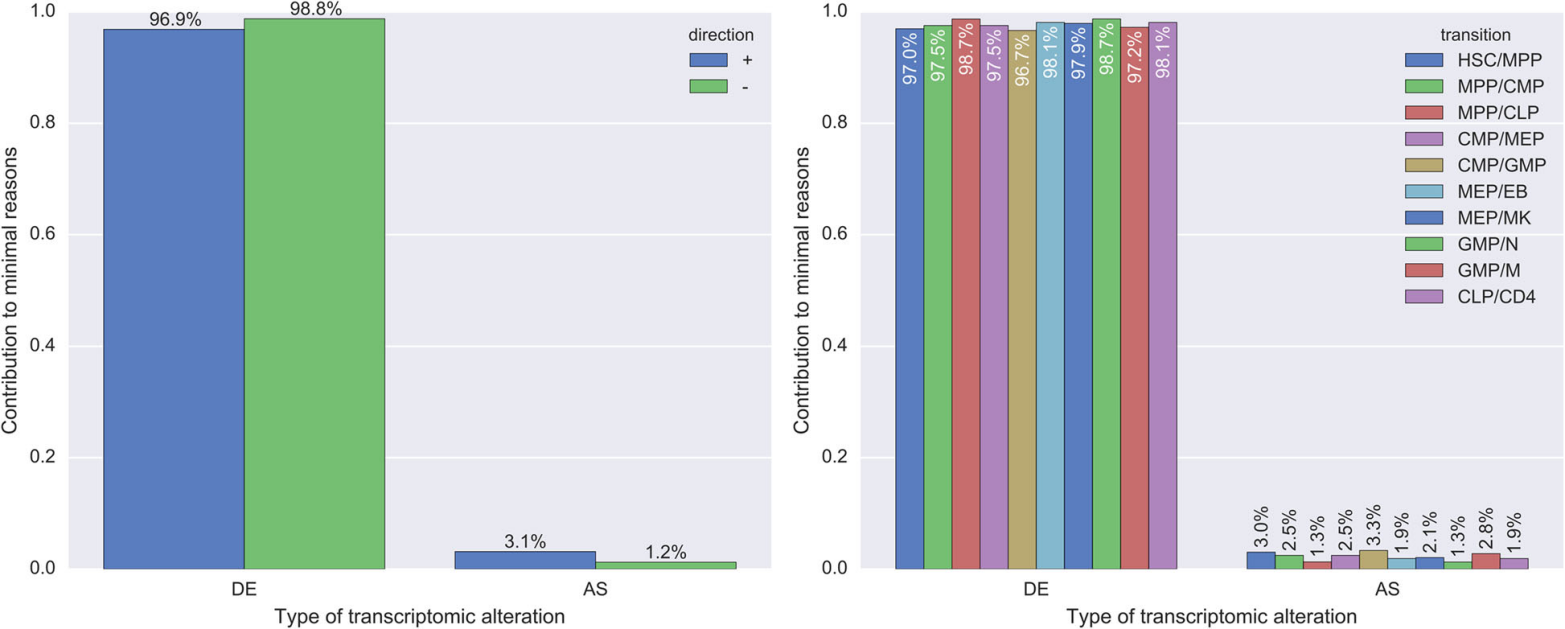
Distributions of alteration types for the minimum amount of explanatory reasons for rewiring events. Shown is the contribution of the two elementary types of conceivable protein alterations in PPICompare, DE and AS, to the solutions of the optimization regarding the small sets of likely changes that explain all rewiring. The contributions are normalized by their direction (*left plot*) or by their proportion in individual transitions (*right plot*). In the *left plot*, “+” (*blue*) means emerging interactions and “-” (*green*) means vanishing interactions

Figure 6 outlines the contributions of the two elementary modes of transcriptomic alterations per protein, DE and AS, to the individual sets and altogether. Also in the reduced set, most of the deregulation events were driven by DE. Yet, the overall proportion of AS was about twice as large as in the comparisons shown previously (in each transition at least 1.3%). Also, the fraction of AS was larger among emerging interactions. The usage of alternative protein isoforms was equally important in all transitions we analyzed.

#### Important alternative splicing events are found in proteins broadly associated with transcriptional control

To assess the functional scope of alternative transcript usage, we submitted the set of all 134 proteins which underwent AS in the sets of most relevant events in any transition to enrichment analysis using the DAVID webservice [65] (see “Methods” section).

DAVID characterized the gene set to be preferentially located in the nucleus (e.g., “nucleoplasm” 2.6 fold enriched), and preferentially concerned with the organization and regulation of chromatin (e.g., “chromatin organization” 3.7 fold enriched and “chromatin modification” 3.2 fold enriched) and with transcriptional regulation (e.g., “DNA binding” 1.9 fold enriched and “transcriptional regulation” 1.9 fold enriched). The family of Basic-Leucine zipper transcription factors seemed to be especially relevant (e.g., “Basic-leucine zipper (bZIP) transcription factor” 9.8 fold enriched, but not significant after adjustment). Further enriched clusters involved post-translational regulatory mechanisms like ubiquitination and related processes (e.g., “Ubl conjugation pathway" 3.8 fold enriched). A detailed listing of all results is provided in Additional file 8: Table S7.

The accumulation of such terms in the altered interaction partners points to the combinatorial and synergistic control of transcription, which is of central importance in all critical developmental circuits in eukaryotes [96–99]. This specificity is of special interest because individual interactions between different transcription factors or transcription factors and cofactors seem to be deliberately switched in a targeted way by AS although both factors are expressed in the cell.

#### Interactions between proteins in the reduced set are likely connectors of functional modules

Co-deregulation of proteins in the small set of changes could hint at important coregulated processes. We started to inspect this possibility by evaluating significantly rewired interactions among proteins of the reduced set in the same fashion as for the general case of simultaneous deregulation. The results are also visualized in Additional file 1: Figure S2.

Interactions altered by those events are associated with more reference protein complexes than the network average but with fewer than the co-deregulated events. They did not differ from co-deregulation events concerning the similarity of processes and colocalization. Whereas the functional similarity was only slightly increased (median GO func. similarity increased by 6%, two-sided Wilcoxon rank-sum test *p*<0.02), there was a striking increase in the betweenness values compared to simultaneous deregulation (median betweenness increased by 31%, two-sided Wilcoxon rank-sum test *p*<10^−78^). The betweenness values were even significantly higher than those of rewiring events for which consistently only one protein was deregulated (median betweenness increased by 13%, two-sided Wilcoxon rank-sum test *p*<10^−69^). This speaks against a possible intramodular role of such interactions in the interactome, but rather hints at a function as intermodular connectors between functional modules. Such connections are very important in signaling, for example, and their dysregulation can be crucial [100]. In fact, the interactions between proteins in the reduced set were enriched in signaling pathways for all developmental transitions (see Additional file 9: Table S8). The apoptosis-relevant interaction of Bcl-2 (BCL2) with the Bcl-2 modifying factor (BMF) [101], for example, emerges in the transition HSC →MPP and is an interaction between proteins of the reduced set (first tab Additional file 9: Table S8).

Complementing this, we used the respective sets of emerging and vanishing interactions individually to determine direction-consistent connected components (CCs) among the reduced protein sets in each transition. The results are listed in Additional file 10: Table S9. Although there existed very large CCs among those interactions (including up to 2,005 proteins in GMP →N, for example), even the large CCs contained comparably few (at most 27) and rather small known CORUM complexes (the largest complex overlapping a CC contained 5 proteins). Within functional modules, one would rather expect that deregulated CCs would preferentially coincide with complexes, though.

#### The reduced set of affected proteins is representative to blood development

In our study, significant rewiring events can be expected to affect proteins that are related to hematopoiesis. We examined this hypothesis by testing how likely it was to sample at least a certain amount of proteins deemed relevant in this context from the reference PPIN by chance. The importance of proteins in that regard was classified according to protein sets that we compiled from GO annotation data and regulatory data from TRRUST [67] (see “Methods” section for details).

We first checked for overrepresentation of hematopoiesis proteins and the regulatory targets of hematopoietic transcription factors. The latter ones were included to also account for proteins that are not obviously associated with hematopoiesis, but that are equally probable to be deregulated due to their direct dependency on regulators of blood development. The set of all proteins affected by rewiring events was highly enriched for those proteins across all transitions (for all transitions *p*<10^−5^, see left half of first sheet in Additional file 11: Table S10). Except for the transition MPP →CMP, the reduced set of deregulated proteins always contained in all other transitions significantly more of those relevant proteins than expected by chance (for all other transitions *p*<0.022, see right half of first sheet in Additional file 11: Table S10).

Similar results were obtained for the set of hematopoiesis proteins without the targets (see second sheet in Additional file 11: Table S10 for details).

#### Known hematopoietic transcription factors are among the drivers of rewiring events

Then, we investigated if known hematopoietic transcription factors were rewired more often than expected by chance and if targets of certain transcription factors were overrepresented in the two protein sets determined (see “Methods” section for details).

Whereas the complete set of proteins involved in rewiring events was highly enriched in hematopoietic transcription factors (for all transitions *p*<3∗10^−4^), this was mostly not the case for the reduced set of proteins (see third sheet in Additional file 11: Table S10). Examples of such rewiring events are discussed below.

Likewise, we found an enrichment of transcription factor targets in the complete set for all transitions. In all but one case this even included known hematopoiesis regulators (see left half of fourth sheet in Additional file 11: Table S10). Again, enrichment was only reported in four transitions for the reduced protein sets (see right half of fourth sheet in Additional file 11: Table S10). Thus, while the optimization procedure can help to effectively decrease the number of proteins of interest, depending on the task at hand the reduction may come along with a loss of information.

Transcription factors for which targets were overrepresented in different developmental transitions are listed in Additional file 11: Table S10.

### Consequences of rewiring during blood development

**Fig. 7:**
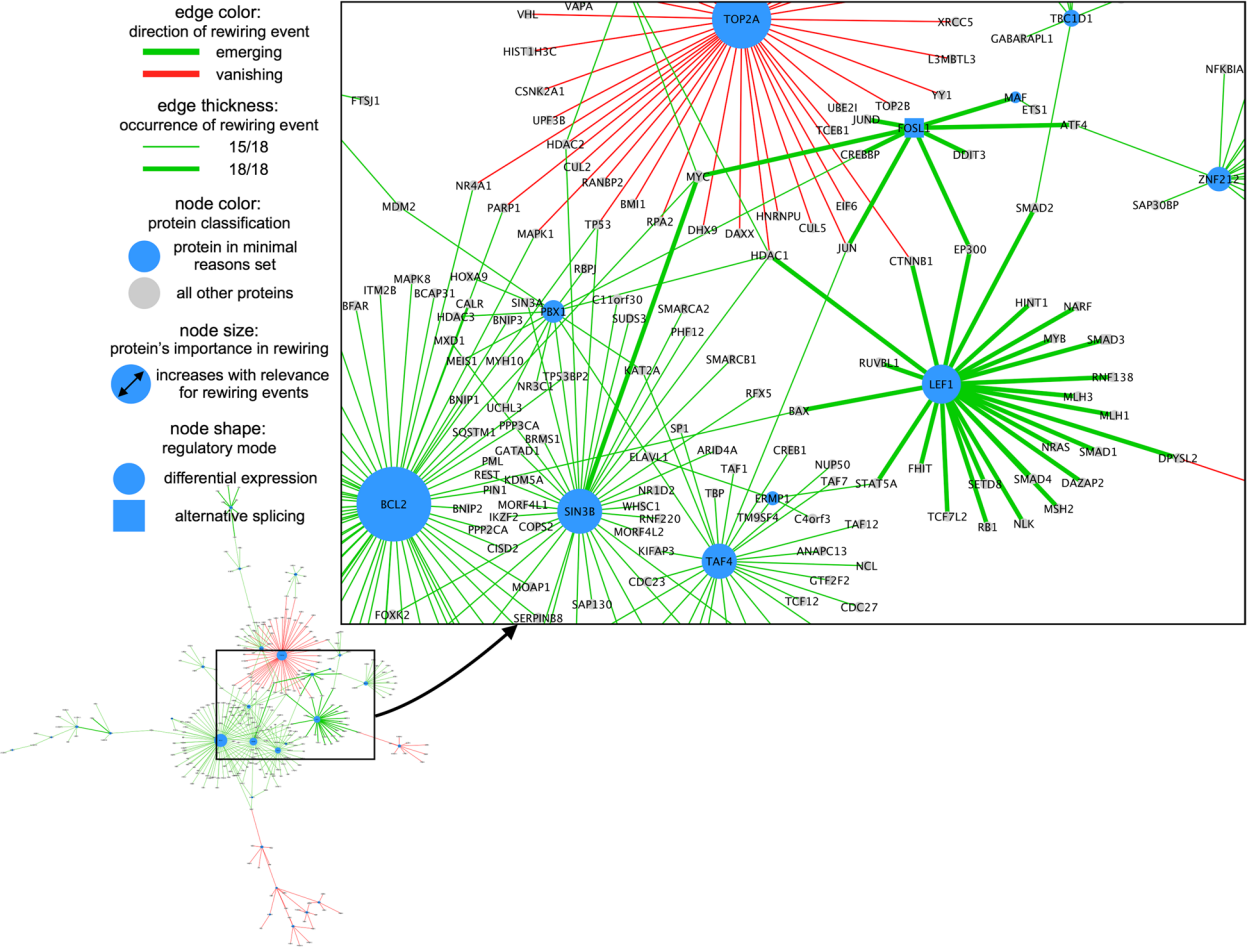
HSC →MPP rewiring events in Cytoscape. We visualized the differential network of the transition HSC →MPP in Cytoscape 3.3 [31] using the default output files of PPICompare. The nodes depict all proteins affected by significant rewiring events. All proteins (internally Uniprot accessions) are displayed with their associated gene’s name. Proteins that belong to the “small set of likely changes” are colored *blue*. The size of nodes increases with their importance score as described in the “Methods” section. Furthermore, protein nodes with a rectangular shape were solely deregulated by AS (here: FOSL1). *Green edges* depict emerging interactions and *red edges* the vanishing ones. The edge thickness indicates how often the event was observed throughout the pairwise comparisons (here either in 15 or in 18 of 18 comparisons). Here, only the largest connected component of the differential network is shown (*lower left*)

At last, we took a brief look into which interactions were changed. The output files of PPICompare are formatted as node- and edge-attribute tables to enable seamless support of network visualization tools such as Cytoscape. Figure 7 shows an illustration of the resulting differential network for the transition HSC →MPP whereby the dense central region is enlarged. Remarkably, this highly connected part of the network is characterized by changes to the interatome between different transcription factors and between transcription factors and cofactors. Such assemblies of transcriptional regulators indeed often have a pivotal role in the context of developmental control [96–99]. Thus, we will focus our attention on this subset of proteins and discuss some of the rewiring events involving proteins considered most relevant by the internal optimization of PPICompare (blue nodes in the visualization).

The transcription factor Fos-related antigen 1 (FOSL1) is a prime example for alternative transcript usage. Upon transition from HSCs to MPPs, its most abundant transcript was switched from ENST00000448083 to ENST00000312562 in every between-group comparison. This shift resulted in the inclusion of a basic-leucine zipper domain (PF00170) which is needed for any dimerization of the protein and thus enabled formation of several new interactions to other regulatory proteins. Among those were coactivator proteins like the (histone) acetyltransferases p300 (EP300) and CREB-binding protein (CREBBP) which are both important integrators of regulatory signals in the hematopietic and other developmental systems [102]. Since such proteins are ubiquitously expressed in all cells, a sole analysis of differential expression would not have been able to detect a difference in that regard between HSCs and MPPs. Interactions of FOSL1 with other transcription factors that were viable after splicing involved c-Jun (JUN), Jun-D (JUND), c-Maf (MAF) or Activating Transcription Factor 4 (ATF4). Together with factors from the Fos-family, these are exchangeable constituents of the transcription factor complex AP-1 and as such control processes including proliferation, differentiation and apoptosis [103, 104]. Further emerging interactions to transcription factors included binding to DNA damage-inducible transcript 3 (DDIT3), that is involved in response to cellular stress, and c-Myc (MYC). Besides its general implication in processes such as cell division, apoptosis, cellular growth, angiogenesis and differentiation, c-Myc is specifically concerned with the balance of self-renewal and differentiation of HSCs [105].

Lymphoid enhancer-binding factor 1 (LEF1) is another protein that changed its expression state in each single comparison and possesses regulatory capabilities in developmental processes beyond the lymphoid lineage [106]. The PPICompare results help in explaining how LEF1-binding may affect its targeted sequence regions mechanistically in MPPs compared to HSCs. Facilitated by the detected differential recruitment of various histone modifying proteins (EP300, HDAC1, SETD8), it could act as the DNA-binding factor for chromatin remodeling events in MPPs, for example. Also, LEF1 may form complexes with *β*-catenin (CTNNB1), T-Cell Factor 4 (TCF7L2), and other proteins (HINT1, RUVBL1) implicated in Wnt/ *β*-catenin signaling, a crucial developmental pathway [107–109]. It may also bind to c-Myb (MYB), a transcription factor controlling regulation of hematopoietic progenitors [110]. Moreover, its abundance in MPPs enabled interactions with the Bcl-2 associated X protein (BAX), which also binds to the important apoptosis regulator Bcl-2 (BCL2). The expression of the latter was upregulated here when hematopoietic progenitor cells become more commited. As correctly determined by PPICompare, Bcl-2 has plenty of new interaction partners in MPPs and thereby ensures a balance of complexes with pro- and antiapoptotic influence (besides BAX: BCL2L1, BAK1, both not visible in figure) [111].

Another upregulated protein deemed important by us was the adaptor protein Sin3b (SIN3B) which facilitates the association of other proteins to epigenetic silencers (REST, HCDA2). Although it apparently did not exert this function in HSCs, it provided c-Myc (MYC) with this capability after the progression to a progenitor cell and furthermore enabled a repressive function of the important hematopoietic transcription factor Helios (IKZF2) of the Ikaros-family [112].

Besides those examples for transcriptional control in HSC →MPP, Additional file 12: Table S11 lists all pathways that are affected by rewiring events. We grouped the events into changes to interactions that were shared between transitions or those exclusive to a certain transition at a developmental branching point.

## Conclusion

Combining PPIXpress and PPICompare enabled us to investigate the dynamics of cause and consequence within the human protein interactome during developmental branching and progression to the extent that this is reflected by transcript expression data. In principle, one can easily detect alterations to any pathway or changes to functional protein complexes, like those concerned with transcriptional regulation. Furthermore, the provided software can aid in suggesting promising targets for the development of new PPI inhibitors, an emerging class of molecules in drug discovery [113]. Beside the general genome-wide trends studied here, the presented pipeline is equally powerful to address very specific questions about rewiring of protein interactions.

## Additional files

Additional file 1 Supplementary information. This PDF contains supplementary results, references and **Figures S1–S5** that are not included in the main text. (PDF 8340 kb)

Additional file 2 **Table S1.** Interactome sizes per cell type for all TPM thresholds examined. Shown are the average number of proteins and the average number of interactions as well as their standard deviations for the constructed protein interaction networks for different discretization thresholds. (XLSX 89.5 kb)

Additional file 3 **Table S2.** Occurrence of each considered type of transcriptomic alteration per transition. Shown are the number of rewiring events for all observed types of transcriptomic alterations when only exclusive causes were considered. (XLSX 9.79 kb)

Additional file 4 **Table S3.** Pathway enrichment analysis of rewiring events controlled by different types of regulation. Listed are pathways for which annotated interactions could be associated with rewiring events across all transitions that were caused by a certain type of regulation. The exact methodology is described in the “Methods” section. In all tables, significantly enriched pathway terms (adj.*p*<0.05) were marked in green. Very small *p*-values were reported as 0.0 due to numerical reasons (computed in Python using SciPy version 0.17 [114]). (XLSX 168 kb)

Additional file 5 **Table S4.** Association of rewiring events with protein complexes. Listed are all rewiring events that affected at least one human CORUM complex [56] separated by transition, type of regulation, and direction. (XLSX 193 kb)

Additional file 6 **Table S5.** Pathway enrichment analysis of rewiring events controlled by different types of regulation separated by transition and direction. Listed are pathways for which annotated interactions could be associated with rewiring events that were caused in a certain transition in a certain direction by a certain type of regulation. The exact methodology is described in the “Methods” section. Significantly enriched pathway terms (adj.*p*<0.05) were marked in green. Very small *p*-values were reported as 0.0 due to numerical reasons (computed in Python using SciPy version 0.17 [114]). (XLSX 455 kb)

Additional file 7 **Table S6.** Sizes of resulting small sets of likely changes in the transcriptome that explain the rewiring in each transition. Listed are the sizes of the resulting small sets of likely changes in the transcriptome that explain the rewiring in each transition, the overall number of rewired interactions for comparison, as well as the number of proteins that are affected by the rewiring. (XLSX 455 kb)

Additional file 8 **Table S7.** DAVID enrichment analysis of AS-driven deregulation events among all sets of likely changes to the transcriptome across all transitions. Results of the analysis were converted to an Excel sheet without further changes. The exact methodology is described in the “Methods” section. (XLSX 101 kb)

Additional file 9 **Table S8.** Pathway enrichment analysis of rewiring events between proteins of the reduced set. Listed are pathways for which annotated interactions could be associated with rewiring events between proteins of the reduced set that were caused in a certain transition. The exact methodology is described in the “Methods” section. Significantly enriched pathway terms (adj.*p*<0.05) were marked in green. Very small *p*-values were reported as 0.0 due to numerical reasons (computed in Python using SciPy version 0.17 [114]). (XLSX 114 kb)

Additional file 10 **Table S9.** Connected components within the reduced set of rewired proteins. Listed are all connected components (CCs) of the direction-specific subnetworks of the reference PPIN (up- and downregulated interactions) defined by the reduced set of rewired proteins. We only included CCs spanning at least 3 proteins. For each CC, we report the number of proteins that were members of the component, the direction of the regulation, and which CORUM complexes were completely included in the component. The size of the respective complexes is given in brackets. (XLSX 16.5 kb)

Additional file 11 **Table S10.** Enrichment of hematopoiesis-specific protein-sets. Listed are the results of hematopoiesis-specific protein-set enrichment analyses for each developmental transition. Query protein-sets were the set of all proteins in the differential network (termed “all”) and the reduced set derived by the internal optimization procedure (termed “reduced”) in the respective transition. We tested the enrichment of the query sets regarding hematopoiesis proteins and the regulatory targets of hematopoietic transcription factors (first sheet, abbreviated as “Hemato. proteins + reg. targets”), hematopoiesis proteins (second sheet, abbreviated as “Hemato. proteins”), and hematopoietic transcription factors (third sheet, abbreviated as “Hemato. TFs”). Furthermore, we determined which transcription factors (not restricted to hematopoietic transcription factors) had more regulatory targets than expected by chance in the queried set (fourth sheet, abbreviated as “Enriched hemato. TF”). The exact methodology is described in the “Methods” section. Here, significance was reported if adj.*p*<0.05. In all tables, test cases that were not significant ((adj.)*p*≥0.05) were highlighted in red. Very small *p*-values were reported as 0.0 due to numerical reasons (computed in Python using SciPy version 0.17 [114]). (XLSX 15.5 kb)

Additional file 12 **Table S11.** Pathway enrichment analysis of rewiring events grouped into shared and exclusive events in each developmental branching point. Listed are pathways for which annotated interactions could be associated with rewiring events that were shared between transitions or exclusive to a transition at a developmental branching point. The exact methodology is described in the “Methods” section. Significantly enriched pathway terms (adj.*p*<0.05) were marked in green. Very small *p*-values were reported as 0.0 due to numerical reasons (computed in Python using SciPy version 0.17 [114]). (XLSX 300 kb)

## Abbreviations

AS: alternative splicing
BP: Biological process
CMP: Common myeloid progenitor
CLP: Common lymphoid progenitor
CD4: Naive CD4 T cells
CC: Cellular compartment
DE: Differential (gene) expression
EB: Erythroblast
ESEA: Edge Set Enrichment Analysis
GMP: Granulocyte monocyte progenitor
HSC: Hematopoetic stem cell
HPM: Human Proteome Map
MPP: Multipotent progenitor
MEP: Megakaryocyte erythrocyte progenitor
MK: Megakaryocyte
M: Monocyte
MF: Molecular function
N: Neutrophil
PPIN: Protein-protein interaction network
PPI: Protein-protein interaction
TPM: Transcripts per million

## References

[1] Snider J, Kotlyar M, Saraon P, Yao Z, Jurisica I, Stagljar I (2015). Fundamentals of protein interaction network mapping. Mol Syst Biol.

[2] Vidal M, Cusick ME, Barabasi AL (2011). Interactome networks and human disease. Cell.

[3] Yeger-Lotem E, Sharan R (2015). Human protein interaction networks across tissues and diseases. Front Genet.

[4] Zhong Q, Simonis N, Li QR, Charloteaux B, Heuze F, Klitgord N (2009). Edgetic perturbation models of human inherited disorders. Mol Syst Biol.

[5] Sahni N, Yi S, Taipale M, Fuxman Bass JI, Coulombe-Huntington J, Yang F (2015). Widespread macromolecular interaction perturbations in human genetic disorders. Cell.

[6] Grossmann A, Benlasfer N, Birth P, Hegele A, Wachsmuth F, Apelt L, Stelzl U (2015). Phospho-tyrosine dependent protein-protein interaction network. Mol Syst Biol.

[7] Bossi A, Lehner B (2009). Tissue specificity and the human protein interaction network. Mol Syst Biol.

[8] Lopes TJ, Schaefer M, Shoemaker J, Matsuoka Y, Fontaine JF, Neumann G (2011). Tissue-specific subnetworks and characteristics of publicly available human protein interaction databases. Bioinformatics.

[9] Sinha A, Nagarajaram HA (2014). Nodes occupying central positions in human tissue specific PPI networks are enriched with many splice variants. Proteomics.

[10] Song J, Wang Z, Ewing RM (2014). Integrated analysis of the Wnt responsive proteome in human cells reveals diverse and cell-type specific networks. Mol Biosyst.

[11] Barshir R, Shwartz O, Smoly IY, Yeger-Lotem E (2014). Comparative analysis of human tissue interactomes reveals factors leading to tissue-specific manifestation of hereditary diseases. PLoS Comput Biol.

[12] Lage K, Hansen NT, Karlberg EO, Eklund AC, Roque FS, Donahoe PK (2008). A large-scale analysis of tissue-specific pathology and gene expression of human disease genes and complexes. Proc Natl Acad Sci U S A.

[13] Magger O, Waldman YY, Ruppin E, Sharan R (2012). Enhancing the prioritization of disease-causing genes through tissue specific protein interaction networks. PLoS Comput Biol.

[14] Ideker T, Krogan NJ (2012). Differential network biology. Mol Syst Biol.

[15] de la Fuente A (2010). From ’differential expression’ to ’differential networking’ - identification of dysfunctional regulatory networks in diseases. Trends Genet.

[16] Han J, Shi X, Zhang Y, Xu Y, Jiang Y, Zhang C (2015). ESEA: Discovering the Dysregulated Pathways based on Edge Set Enrichment Analysis. Sci Rep.

[17] Ji J, Yuan Z, Zhang X, Xue F (2016). A powerful score-based statistical test for group difference in weighted biological networks. BMC Bioinformatics.

[18] Reverter A, Ingham A, Lehnert SA, Tan SH, Wang Y, Ratnakumar A (2006). Simultaneous identification of differential gene expression and connectivity in inflammation, adipogenesis and cancer. Bioinformatics.

[19] Gill R, Datta S, Datta S (2010). A statistical framework for differential network analysis from microarray data. BMC Bioinformatics.

[20] Ruan D, Young A, Montana G (2015). Differential analysis of biological networks. BMC Bioinformatics.

[21] Landeghem SV, Parys TV, Dubois M, Inze D, de Peer YV (2016). Diffany: an ontology-driven framework to infer, visualise and analyse differential molecular networks. BMC Bioinformatics.

[22] Yang X, Coulombe-Huntington J, Kang S, Sheynkman GM, Hao T, Richardson A (2016). Widespread expansion of protein interaction capabilities by alternative splicing. Cell.

[23] Corominas R, Yang X, Lin GN, Kang S, Shen Y, Ghamsari L (2014). Protein interaction network of alternatively spliced isoforms from brain links genetic risk factors for autism. Nat Commun.

[24] Ellis JD, Barrios-Rodiles M, Colak R, Irimia M, Kim T, Calarco JA (2012). Tissue-specific alternative splicing remodels protein-protein interaction networks. Mol Cell.

[25] Buljan M, Chalancon G, Eustermann S, Wagner GP, Fuxreiter M, Bateman A (2012). Tissue-specific splicing of disordered segments that embed binding motifs rewires protein interaction networks. Mol Cell.

[26] Will T, Helms V (2016). PPIXpress: construction of condition-specific protein interaction networks based on transcript expression. Bioinformatics.

[27] Orkin SH, Zon LI (2008). Hematopoiesis: an evolving paradigm for stem cell biology. Cell.

[28] Martens JH, Stunnenberg HG (2013). BLUEPRINT: mapping human blood cell epigenomes. Haematologica.

[29] BLUEPRINT Epigenome Project 7th Data Release. 2015. http://dx.doi.org/10.6019/blueprint_20150910. Accessed 22 Jan 2016.

[30] Chen L, Kostadima M, Martens JH, Canu G, Garcia SP, Turro E (2014). Transcriptional diversity during lineage commitment of human blood progenitors. Science.

[31] Shannon P, Markiel A, Ozier O, Baliga NS, Wang JT, Ramage D (2003). Cytoscape: a software environment for integrated models of biomolecular interaction networks. Genome Res.

[32] Gallo CA, Cecchini RL, Carballido JA, Micheletto S, Ponzoni I (2016). Discretization of gene expression data revised. Brief Bioinformatics.

[33] Levandowsky M, Winter D (1971). Distance between sets. Nature.

[34] Benjamini Y, Hochberg Y (1995). Controlling the False Discovery Rate: A Practical and Powerful Approach to Multiple Testing, Journal of the Royal Statistical Society. Ser B (Methodol).

[35] Young NE (2008). Greedy set-cover algorithms. Encyclopedia of Algorithms.

[36] Chvatal V (1979). A greedy heuristic for the set-covering problem. Math Oper Res.

[37] Lara-Astiaso D, Weiner A, Lorenzo-Vivas E, Zaretsky I, Jaitin DA, David E (2014). Chromatin state dynamics during blood formation. Science.

[38] Bock C, Beerman I, Lien WH, Smith ZD, Gu H, Boyle P (2012). DNA methylation dynamics during in vivo differentiation of blood and skin stem cells. Mol Cell.

[39] Novershtern N, Subramanian A, Lawton LN, Mak RH, Haining WN, McConkey ME (2011). Densely interconnected transcriptional circuits control cell states in human hematopoiesis. Cell.

[40] Doulatov S, Notta F, Laurenti E, Dick JE (2012). Hematopoiesis: a human perspective. Cell Stem Cell.

[41] Yamamoto R, Morita Y, Ooehara J, Hamanaka S, Onodera M, Rudolph KL (2013). Clonal analysis unveils self-renewing lineage-restricted progenitors generated directly from hematopoietic stem cells. Cell.

[42] Perie L, Duffy KR, Kok L, de Boer RJ, Schumacher TN (2015). The branching point in erythro-myeloid differentiation. Cell.

[43] Nimmo RA, May GE, Enver T (2015). Primed and ready: understanding lineage commitment through single cell analysis. Trends Cell Biol.

[44] Adolfsson J, Mansson R, Buza-Vidas N, Hultquist A, Liuba K, Jensen CT (2005). Identification of Flt3+ lympho-myeloid stem cells lacking erythro-megakaryocytic potential a revised road map for adult blood lineage commitment. Cell.

[45] Notta F, Zandi S, Takayama N, Dobson S, Gan OI, Wilson G (2016). Distinct routes of lineage development reshape the human blood hierarchy across ontogeny. Science.

[46] Li B, Dewey CN (2011). RSEM: accurate transcript quantification from RNA-Seq data with or without a reference genome. BMC Bioinformatics.

[47] Conesa A, Madrigal P, Tarazona S, Gomez-Cabrero D, Cervera A, McPherson A (2016). A survey of best practices for RNA-seq data analysis. Genome Biol.

[48] Li B, Ruotti V, Stewart RM, Thomson JA, Dewey CN (2010). RNA-Seq gene expression estimation with read mapping uncertainty. Bioinformatics.

[49] Calderone A, Castagnoli L, Cesareni G (2013). mentha: a resource for browsing integrated protein-interaction networks. Nat Methods.

[50] Bateman A, Martin MJ, O’Donovan C, Magrane M, Apweiler R, Alpi E (2015). UniProt: a hub for protein information. Nucleic Acids Res.

[51] Yates A, Akanni W, Amode MR, Barrell D, Billis K, Carvalho-Silva D (2016). Ensembl 2016. Nucleic Acids Res.

[52] Mosca R, Ceol A, Stein A, Olivella R, Aloy P (2014). 3did: a catalog of domain-based interactions of known three-dimensional structure. Nucleic Acids Res.

[53] Finn RD, Miller BL, Clements J, Bateman A (2014). iPfam: a database of protein family and domain interactions found in the Protein Data Bank. Nucleic Acids Res.

[54] Kim MS, Pinto SM, Getnet D, Nirujogi RS, Manda SS, Chaerkady R (2014). A draft map of the human proteome. Nature.

[55] Gray KA, Yates B, Seal RL, Wright MW, Bruford EA (2015). Genenames.org: the HGNC resources in 2015. Nucleic Acids Res.

[56] Ruepp A, Waegele B, Lechner M, Brauner B, Dunger-Kaltenbach I, Fobo G (2010). CORUM: the comprehensive resource of mammalian protein complexes–2009. Nucleic Acids Res.

[57] Blake JA, Christie KR, Dolan ME, Drabkin HJ, Hill DP, Ni L (2015). Gene Ontology Consortium: going forward. Nucleic Acids Res.

[58] Yu G, Li F, Qin Y, Bo X, Wu Y, Wang S (2010). GOSemSim: an R package for measuring semantic similarity among GO terms and gene products. Bioinformatics.

[59] Carlson M. org.Hs.eg.db: Genome Wide Annotation for Human. R package version 3.2.3. http://bioconductor.org/packages/release/data/annotation/html/org.Hs.eg.db.html.

[60] Hagberg AA, Schult DA, Swart PJ. Exploring network structure, dynamics, and function using NetworkX. In: Proceedings of the 7th Python in Science Conference (SciPy2008). Pasadena: 2008. p. 11–15.

[61] Kanehisa M, Sato Y, Kawashima M, Furumichi M, Tanabe M (2016). KEGG as a reference resource for gene and protein annotation. Nucleic Acids Res.

[62] Fabregat A, Sidiropoulos K, Garapati P, Gillespie M, Hausmann K, Haw R (2016). The Reactome pathway Knowledgebase. Nucleic Acids Res.

[63] Khatri P, Draghici S (2005). Ontological analysis of gene expression data: current tools, limitations, and open problems. Bioinformatics.

[64] Subramanian A, Tamayo P, Mootha VK, Mukherjee S, Ebert BL, Gillette MA (2005). Gene set enrichment analysis: a knowledge-based approach for interpreting genome-wide expression profiles. Proc Natl Acad Sci U S A.

[65] Huang daW, Sherman BT, Lempicki RA (2009). Systematic and integrative analysis of large gene lists using DAVID bioinformatics resources. Nat Protoc.

[66] Binns D, Dimmer E, Huntley R, Barrell D, O’Donovan C, Apweiler R (2009). QuickGO: a web-based tool for Gene Ontology searching. Bioinformatics.

[67] Han H, Shim H, Shin D, Shim JE, Ko Y, Shin J (2015). TRRUST: a reference database of human transcriptional regulatory interactions. Sci Rep.

[68] Trapnell C (2015). Defining cell types and states with single-cell genomics. Genome Res.

[69] Etzrodt M, Endele M, Schroeder T (2014). Quantitative single-cell approaches to stem cell research. Cell Stem Cell.

[70] Paul F, Arkin Y, Giladi A, Jaitin DA, Kenigsberg E, Keren-Shaul H (2015). Transcriptional heterogeneity and lineage commitment in myeloid progenitors. Cell.

[71] Zhu J, Yamane H, Paul WE (2010). Differentiation of effector CD4 T cell populations (*). Annu Rev Immunol.

[72] Hong T, Xing J, Li L, Tyson JJ (2012). A simple theoretical framework for understanding heterogeneous differentiation of CD4+ T cells. BMC Syst Biol.

[73] Yona S, Jung S (2010). Monocytes: subsets, origins, fates and functions. Curr Opin Hematol.

[74] Cabezas-Wallscheid N, Klimmeck D, Hansson J, Lipka DB, Reyes A, Wang Q (2014). Identification of regulatory networks in HSCs and their immediate progeny via integrated proteome, transcriptome, and DNA methylome analysis. Cell Stem Cell.

[75] Gonzalez-Porta M, Frankish A, Rung J, Harrow J, Brazma A (2013). Transcriptome analysis of human tissues and cell lines reveals one dominant transcript per gene. Genome Biol.

[76] Ezkurdia I, del Pozo A, Frankish A, Rodriguez JM, Harrow J, Ashman K (2012). Comparative proteomics reveals a significant bias toward alternative protein isoforms with conserved structure and function. Mol Biol Evol.

[77] Mele M, Ferreira PG, Reverter F, DeLuca DS, Monlong J, Sammeth M (2015). The human transcriptome across tissues and individuals. Science.

[78] Barbosa-Morais NL, Irimia M, Pan Q, Xiong HY, Gueroussov S, Lee LJ (2012). The evolutionary landscape of alternative splicing in vertebrate species. Science.

[79] Corominas R, Yang X, Lin GN, Kang S, Shen Y, Ghamsari L (2014). Protein interaction network of alternatively spliced isoforms from brain links genetic risk factors for autism. Nat Commun.

[80] Ni JZ, Grate L, Donohue JP, Preston C, Nobida N, O’Brien G (2007). Ultraconserved elements are associated with homeostatic control of splicing regulators by alternative splicing and nonsense-mediated decay. Genes Dev.

[81] Saltzman AL, Kim YK, Pan Q, Fagnani MM, Maquat LE, Blencowe BJ (2008). Regulation of multiple core spliceosomal proteins by alternative splicing-coupled nonsense-mediated mRNA decay. Mol Cell Biol.

[82] Han JD, Bertin N, Hao T, Goldberg DS, Berriz GF, Zhang LV (2004). Evidence for dynamically organized modularity in the yeast protein-protein interaction network. Nature.

[83] Fraser HB (2005). Modularity and evolutionary constraint on proteins. Nat Genet.

[84] Kim PM, Lu LJ, Xia Y, Gerstein MB (2006). Relating three-dimensional structures to protein networks provides evolutionary insights. Science.

[85] Chang X, Xu T, Li Y, Wang K (2013). Dynamic modular architecture of protein-protein interaction networks beyond the dichotomy of ’date’ and ’party’ hubs. Sci Rep.

[86] Narayanan T, Gersten M, Subramaniam S, Grama A (2011). Modularity detection in protein-protein interaction networks. BMC Res Notes.

[87] Dunn R, Dudbridge F, Sanderson CM (2005). The use of edge-betweenness clustering to investigate biological function in protein interaction networks. BMC Bioinformatics.

[88] Shi M, Lin TH, Appell KC, Berg LJ (2009). Cell cycle progression following naive T cell activation is independent of Jak3/common gamma-chain cytokine signals. J Immunol.

[89] Theilgaard-Monch K, Jacobsen LC, Borup R, Rasmussen T, Bjerregaard MD, Nielsen FC (2005). The transcriptional program of terminal granulocytic differentiation. Blood.

[90] van Furth R, Raeburn JA, van Zwet TL (1979). Characteristics of human mononuclear phagocytes. Blood.

[91] Fossati G, Moulding DA, Spiller DG, Moots RJ, White MR, Edwards SW (2003). The mitochondrial network of human neutrophils: role in chemotaxis, phagocytosis, respiratory burst activation, and commitment to apoptosis. J Immunol.

[92] Kramer PA, Ravi S, Chacko B, Johnson MS, Darley-Usmar VM (2014). A review of the mitochondrial and glycolytic metabolism in human platelets and leukocytes: implications for their use as bioenergetic biomarkers. Redox Biol.

[93] Graczyk D, White RJ, Ryan KM (2015). Involvement of RNA Polymerase III in Immune Responses. Mol Cell Biol.

[94] Chiu YH, Macmillan JB, Chen ZJ (2009). RNA polymerase III detects cytosolic DNA and induces type I interferons through the RIG-I pathway. Cell.

[95] Tamassia N, Bazzoni F, Le Moigne V, Calzetti F, Masala C, Grisendi G (2012). IFN-Beta expression is directly activated in human neutrophils transfected with plasmid DNA and is further increased via TLR-4-mediated signaling. J Immunol.

[96] Lelli KM, Slattery M, Mann RS (2012). Disentangling the many layers of eukaryotic transcriptional regulation. Annu Rev Genet.

[97] Hochedlinger K, Plath K (2009). Epigenetic reprogramming and induced pluripotency. Development.

[98] Spitz F, Furlong EE (2012). Transcription factors: from enhancer binding to developmental control. Nat Rev Genet.

[99] Wilson NK, Foster SD, Wang X, Knezevic K, Schutte J, Kaimakis P (2010). Combinatorial transcriptional control in blood stem/progenitor cells: genome-wide analysis of ten major transcriptional regulators. Cell Stem Cell.

[100] Taylor IW, Linding R, Warde-Farley D, Liu Y, Pesquita C, Faria D (2009). Dynamic modularity in protein interaction networks predicts breast cancer outcome. Nat Biotechnol.

[101] Pinon JD, Labi V, Egle A, Villunger A (2008). Bim and Bmf in tissue homeostasis and malignant disease. Oncogene.

[102] Blobel GA (2000). CREB-binding protein and p300: molecular integrators of hematopoietic transcription. Blood.

[103] Shaulian E, Karin M (2002). AP-1 as a regulator of cell life and death. Nat Cell Biol.

[104] Steinmuller L, Cibelli G, Moll JR, Vinson C, Thiel G (2001). Regulation and composition of activator protein 1 (AP-1) transcription factors controlling collagenase and c-Jun promoter activities. Biochem J.

[105] Wilson A, Murphy MJ, Oskarsson T, Kaloulis K, Bettess MD, Oser GM (2004). c-Myc controls the balance between hematopoietic stem cell self-renewal and differentiation. Genes Dev.

[106] Skokowa J, Cario G, Uenalan M, Schambach A, Germeshausen M, Battmer K (2006). LEF-1 is crucial for neutrophil granulocytopoiesis and its expression is severely reduced in congenital neutropenia. Nat Med.

[107] MacDonald BT, Tamai K, He X (2009). Wnt/beta-catenin signaling: components, mechanisms, and diseases. Dev Cell.

[108] Genovese G, Ghosh P, Li H, Rettino A, Sioletic S, Cittadini A (2012). The tumor suppressor HINT1 regulates MITF and beta-catenin transcriptional activity in melanoma cells. Cell Cycle.

[109] Bauer A, Huber O, Kemler R (1998). Pontin52, an interaction partner of beta-catenin, binds to the TATA box binding protein. Proc Natl Acad Sci USA.

[110] Soza-Ried C, Hess I, Netuschil N, Schorpp M, Boehm T (2010). Essential role of c-myb in definitive hematopoiesis is evolutionarily conserved. Proc Natl Acad Sci USA.

[111] Orelio C, Dzierzak E (2007). Bcl-2 expression and apoptosis in the regulation of hematopoietic stem cells. Leuk Lymphoma.

[112] Koipally J, Georgopoulos K (2002). A molecular dissection of the repression circuitry of Ikaros. J Biol Chem.

[113] Scott DE, Bayly AR, Abell C, Skidmore J (2016). Small molecules, big targets: drug discovery faces the protein-protein interaction challenge. Nat Rev Drug Discov.

[114] Jones E, Oliphant T, Peterson P, et al. SciPy: Open source scientific tools for Python. 2001. http://www.scipy.org/. Accessed 07 June 2016.

