# Supplementary material for: Rewiring of the inferred protein interactome during blood development studied with the tool PPICompare
Source: BMC Syst Biol. 2017 Apr 4;11:44. doi: 10.1186/s12918-017-0400-x (PMC5379774; doi:10.1186/s12918-017-0400-x)
Supplement: Supplementary file 1 — Supplementary information. This PDF contains supplementary results, references and Figures S1–S5 that are not included in the main text. (PDF 8340 kb) [file 12918_2017_400_MOESM1_ESM.pdf]

PPICompare
Supplementary Material

T. Will and V. Helms

S1 Supplementary results

S1.1 Tuning the expression threshold parameter

Establishing a fixed expression value that separates silenced from expressed transcripts is a somehow arbitrary choice [4, 8]. Previously, authors used TPM thresholds ranging from slightly above 0.0 TPM [2, 5] to 1.0 TPM [1] in various downstream analyses. To implement a threshold that reflects the actual protein concentrations in the cell, we used mass spectrometry-based proteome abundance data from HPM [6] for guidance. This independent experimental ressource shares information on CD4 and M cells with our constructed blood cell interactomes. Since the samples were derived from the same tissue of origin in both data sources and the annotational coverage was basically the same for these two cell types in HPM and in the reference PPIN (17,294 annotated HGNC protein-coding genes for HPM data and 17,292 proteins in the reference PPIN), we considered the reported proteome abundances and the number of proteins in our PPINs to be sufficiently comparable.

According to the HPM data, 8,140 proteins were detected in CD4 cells and 7,781 proteins in M cells. We compared these values to the number of proteins found in the constructed CD4 and M networks, i.e. the proteins for which the mRNA levels exceeded different expression thresholds (see Suppl. Table S1 for details). The optimal TPM threshold for our purpose was determined as the one with the least absolute deviation between the number of proteins in the networks and the respective references (see Figure S4). This metric is considered robust concerning outliers [3]. With an average deviation of 553 proteins per sample, a threshold of 0.31 TPM gave the best agreement. In this case, the mean number of proteins in the 8 CD4 networks differed only by 117 proteins from the proteomic reference (10% of the standard deviation across samples) and the mean of the 5 monocyte networks differed only by 10 proteins (7.4% of the standard deviation across samples). All further analyses presented below were performed on the protein interaction networks constructed with the HPM-derived threshold of 0.31 TPM.

S1.2 Sample-size dependency of PPICompare results

Subsampling of a reference dataset is a common way to test the robustness of a differential analysis and was frequently conducted in the study of differential expression, see [7, 9, 10], for example. In the same manner, we tested PPICompare on subsets of samples for the transition CLP→CD4 (see Figure 2 in the main text) for which the BLUEPRINT data used contained the largest number of samples (5 samples for CLPs and 8 for CD4 cells). For both cell types we enumerated all subsets with at least 2 samples. We then combined the 26 subsets for CLP and the 247 subsets for CD4 cells to obtain 6,422 pairs of subsamples of the initial data with varying sample size. For all subsets we determined differential interactions among the network samples constructed with HPM-optimal threshold using PPICompare with FDR 0.05. Based on the results of the complete data for CLP→CD4 we calculated the precision (fraction of reference rewirings among all rewirings found by application to the subset) and the recall (fraction of reference rewirings found). From those we derived the balanced F-score (F_1-score) as the harmonic mean of precision and recall. Figure S5 shows these measures as a function

of the number of samples used in the corresponding calculations and the size of the smaller group, respectively. The latter is meant to grasp the effect of unbalanced group sizes.

It was not surprising to find that more data and a better balance generally led to a better overall agreement with the rewiring events detected on the basis of the full set of 13 samples (see F-score in the upper left panel of Figure S5).

Precision increases with the size of the smaller group

For subsets that had a smaller group of equal size, the median precision did often not increase considerably or even decreased with each added sample (see lower left panel of Figure S5). Instead, the precision depended more strongly on the size of the smaller group. This means that a better balance of the sample sizes between the groups was beneficial for the precision when the overall number of samples was fixed. The most remarkable increase of precision was consistently found when subsets with a group that only had 2 samples were compared to subsets that had 3 samples in the smaller group. Then, on average at least 19% (see 9 samples) and up to 34% (see 6 samples) more rewiring events within the results of each subset were found in the results of the complete data. For comparison, the increase from 3 to 4 samples in the smaller group was at most 18% (see 9 samples) and that from 4 to 5 only 15% (see 11 samples). The large number of rewiring events detected (see upper right panel) and a recall level that is similar to that of the more balanced subsets (see lower right panel) suggest that a high group imbalance causes an emphasis on the detection of the individual differences between the subsamples, rather than the more general differences over the group as a whole. As a consequence, more rewiring events were detected but the fraction of those that were significant in the whole dataset was smaller.

Recall increases with the overall number of samples

For the recall, the size of the smaller group had generally a less pronounced direct effect (see lower right panel of Figure S5). For subsets with a small group of only 2 or 3 samples, the increase seemed to happen stepwise. This was best visible for subsets of the data, where the smaller group contained 3 representatives: whereas the main part of the distribution of recall values was in a similar range for 6 to 8 total samples, the recall immediately increased from 9 samples onward to again reach a kind of steady state (see also the number of detected rewiring events in the upper right panel). This observation can be explained by the general implication of pairwise comparison and the strict significance filtering. When comparing pairwise with a small group of size s that includes a single outlier with skewed relevant feature f (say, a change in gene abundance), feature f cannot be recognized in $\frac{1}{s}$ of all cases and may thus not be found of significance anymore. However, with increasing sample size both the resolution of the sampling and the statistical power of the applied test increase, and may then enable passing a significance-threshold barrier.

Although having more data is of course always beneficial, our practical evaluation shows that even with less than half of the data satisfactory results with relatively few false positives could be obtained (average precision 0.78 with only 3 samples per group). We recommend to use PPICompare with at least 3 samples per group, because this yields a robust precision. Consequently, we only examined cell types with at least that much data available (see Methods section).

S2 Supplemental Figures

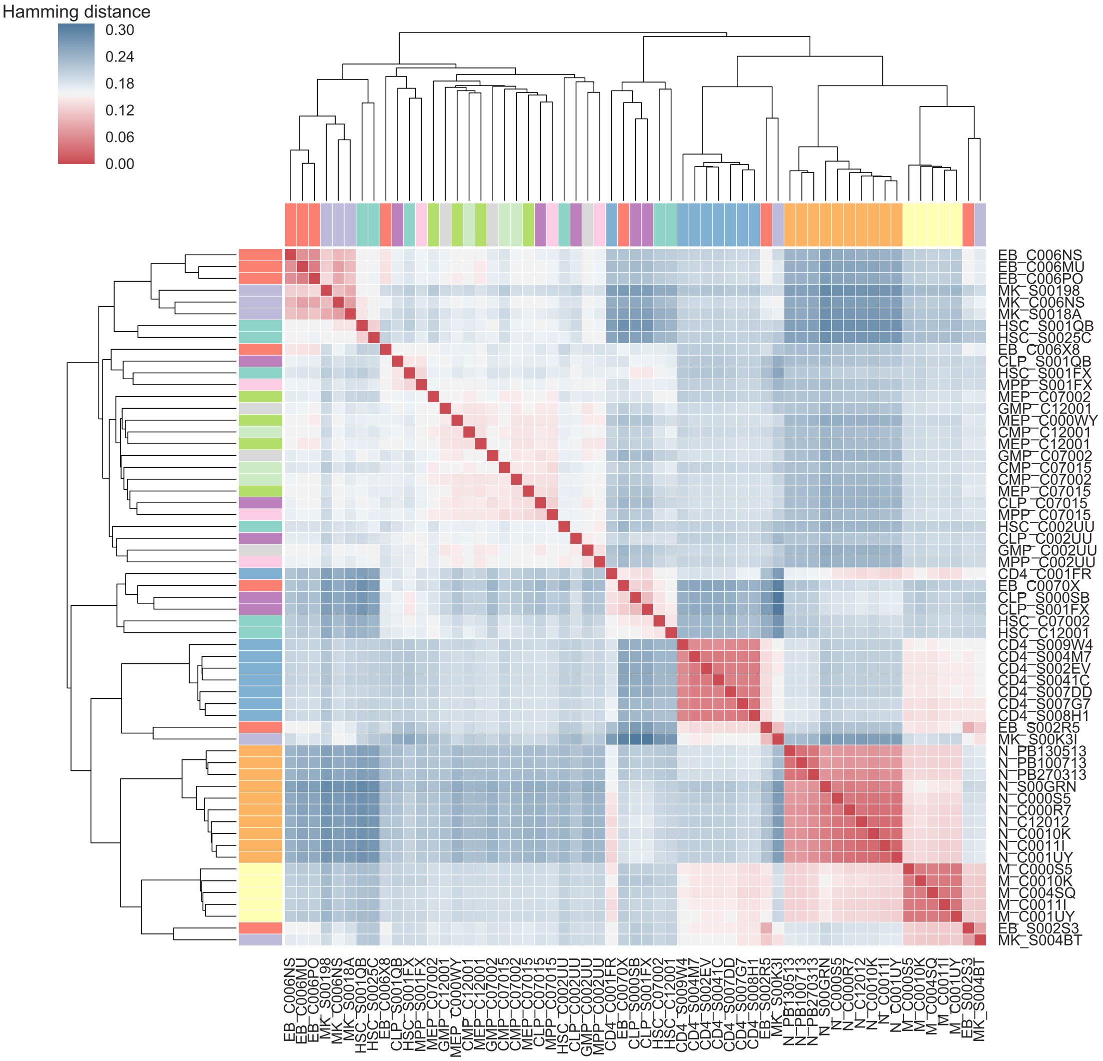

Figure S1: Hierarchical clustering of hematopoiesis cell types. *Results of average linkage clustering (UPGMA) applied to all samples based on the normalized Hamming distance of abundant transcripts discretized using the optimal TPM threshold (boolean vector of transcript abundances concerning all transcripts associated with a Uniprot accession in Ensembl 83). Cell types are additionally distinguished by colored labels.*

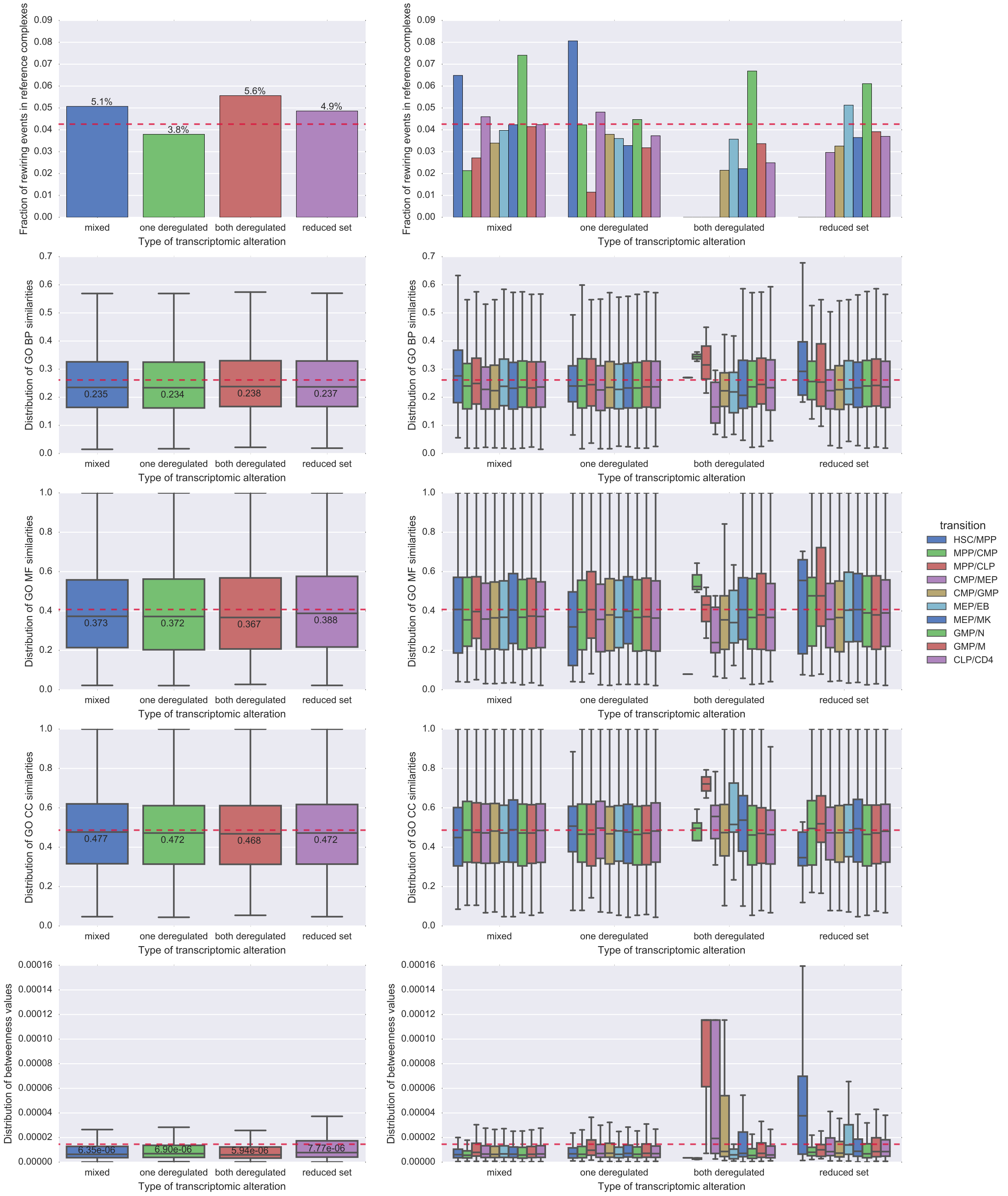

Figure S2: Comparison of rewiring events caused by co-deregulatory and non-co-deregulatory transcriptomic alterations. *We separated all significant rewiring events into four groups of co-deregulatory and non-co-deregulatory types of transcriptomic alterations and examined their behaviour concerning unrelated indicators of modularity. We distinguished between the disjoint groups of rewiring events of mixed cause ("mixed"), those with one deregulated interaction partner ("one deregulated"), and those controlled by simultaneous deregulation of both partners ("both deregulated"). The last category additionally shows the results for rewiring events in which both interaction partners were proteins affected by the small set of likely changes in the transcriptome determined by the internal optimization approach ("reduced set"). The respective classification is introduced in the appropriate paragraphs of the main text. The plots on the left side show the results for all rewiring events affected by this mode of regulation while the plots on the right side depict the results per transition. The dashed red lines always represent the average of the respective measure in the complete reference network. Note that HSC→MPP, MPP→CMP and MPP→CLP had very few (< 5) co-deregulated rewiring events (see also Suppl. Table S2).*

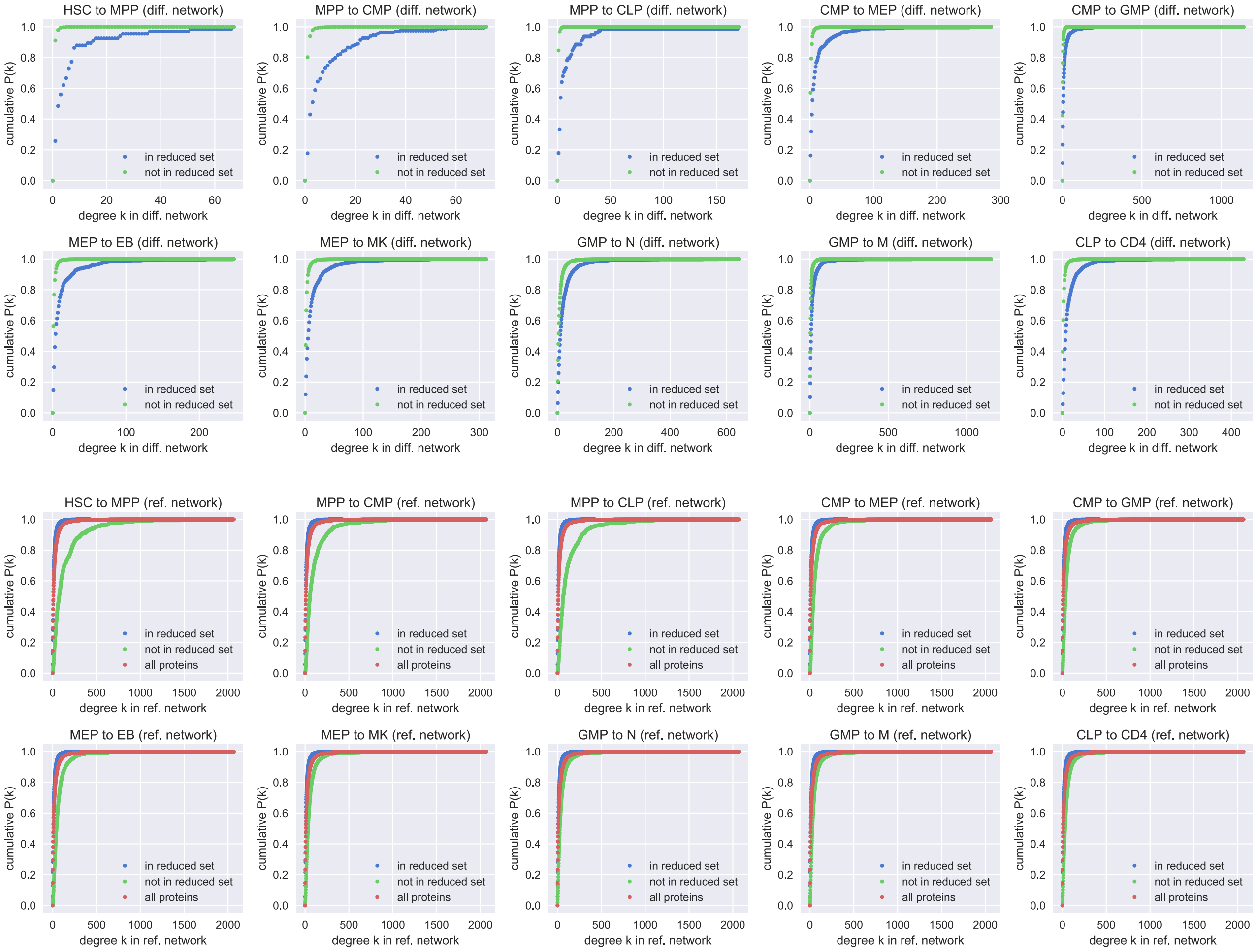

Figure S3: Cumulative degree distributions of rewired proteins. *Cumulative degree distributions of the rewired proteins per transition in the corresponding differential networks (upper half) and of the rewired proteins per transition in the reference protein interaction network (lower half). The rewired proteins are additionally split up into those in the reduced set and the remaining ones, "all proteins" depicts all proteins in the reference network.*

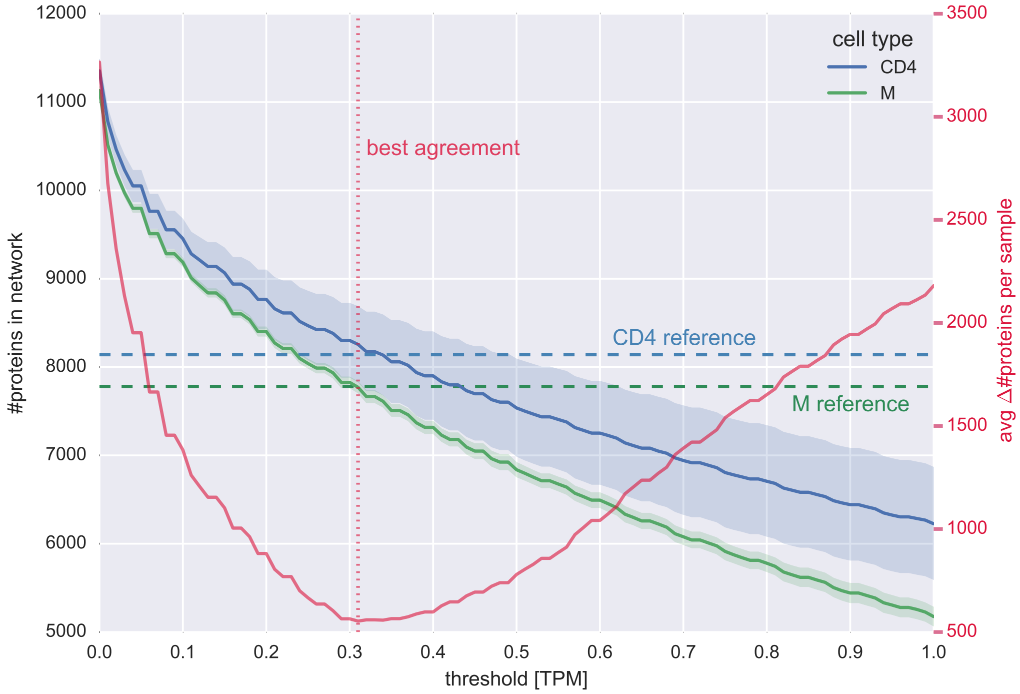

Figure S4: Matching the TPM threshold of the network construction to proteome abundance data. *The number of proteins in the constructed CD4 and M interaction networks across different TPM thresholds (solid blue and green lines with standard deviation) is compared to proteomic reference data (dashed blue and green lines). According to the least absolute derivation (solid red line), 0.31 TPM (dashed red line) gave the best agreement.*

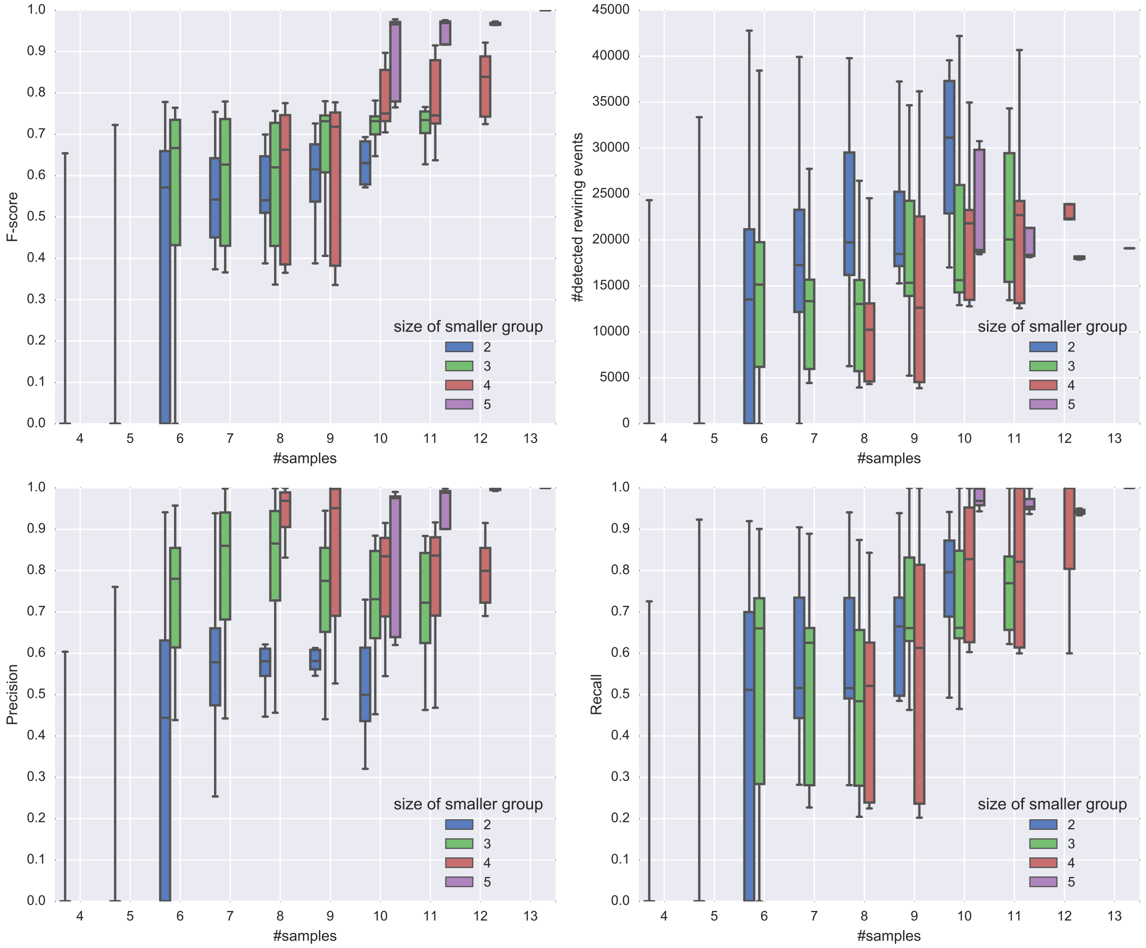

Figure S5: ***Sample-size dependency of PPICompare results for the transition CLP→CD4.*** *We analyzed the rewired interactions found when only a subset of the available data was used and compared their agreement to the result obtained with all 13 samples that was taken as reference and thus set to 1.0.*

References

[1] R. Andersson et al. An atlas of active enhancers across human cell types and tissues. *Nature*, 507(7493):455–461, Mar 2014.

[2] F. Danielsson et al. Assessing the consistency of public human tissue RNA-seq data sets. *Brief. Bioinformatics*, 16(6):941–949, Nov 2015.

[3] Stephen Gorard. Revisiting a 90-year-old debate: the advantages of the mean deviation. *British Journal of Educational Studies*, 53(4):417–430, 2005.

[4] T. Hart et al. Finding the active genes in deep RNA-seq gene expression studies. *BMC Genomics*, 14:778, 2013.

[5] A. Kanitz et al. Comparative assessment of methods for the computational inference of transcript isoform abundance from RNA-seq data. *Genome Biol.*, 16:150, 2015.

[6] M. S. Kim et al. A draft map of the human proteome. *Nature*, 509(7502):575–581, May 2014.

[7] Y. Liu et al. RNA-seq differential expression studies: more sequence or more replication? *Bioinformatics*, 30(3):301–304, Feb 2014.

[8] D. Ramskold et al. An abundance of ubiquitously expressed genes revealed by tissue transcriptome sequence data. *PLoS Comput. Biol.*, 5(12):e1000598, Dec 2009.

[9] F. Rapaport et al. Comprehensive evaluation of differential gene expression analysis methods for RNA-seq data. *Genome Biol.*, 14(9):R95, 2013.

[10] Z. H. Zhang et al. A comparative study of techniques for differential expression analysis on RNA-Seq data. *PLoS ONE*, 9(8):e103207, 2014.
